# Amplitudes of resting-state functional networks – investigation into their correlates and biophysical properties

**DOI:** 10.1016/j.neuroimage.2022.119779

**Published:** 2023-01

**Authors:** Soojin Lee, Janine D. Bijsterbosch, Fidel Alfaro Almagro, Lloyd Elliott, Paul McCarthy, Bernd Taschler, Roser Sala-Llonch, Christian F. Beckmann, Eugene P. Duff, Stephen M. Smith, Gwenaëlle Douaud

**Affiliations:** a Centre for Functional MRI of the Brain (FMRIB), Wellcome Centre for Integrative Neuroimaging, Nuffield Department of Clinical Neurosciences, University of Oxford, UK; b Pacific Parkinson's Research Institute, University of British Columbia, Canada; c Mallinckrodt Institute of Radiology, Washington University Medical School, Washington University in St Louis, USA; d Department of Statistics and Actuarial Science, Simon Fraser University (SFU), Canada; e Department of Biomedicine, Institute of Neurosciences, University of Barcelona, Spain; f Department of Cognitive Neuroscience, Radboud University Medical Centre, Nijmegen, the Netherlands; g Donders Institute for Brain, Cognition and Behaviour, Radboud University Nijmegen, Nijmegen, the Netherlands; h Department of Brain Sciences, Imperial College London, UK Dementia Research Institute, London UK

**Keywords:** Resting-state fMRI, Network amplitude, Dual regression, Temporal synchrony, UK Biobank, GWAS

## Abstract

Resting-state fMRI studies have shown that multiple functional networks, which consist of distributed brain regions that share synchronised spontaneous activity, co-exist in the brain. As these resting-state networks (RSNs) have been thought to reflect the brain's intrinsic functional organization, intersubject variability in the networks’ spontaneous fluctuations may be associated with individuals’ clinical, physiological, cognitive, and genetic traits. Here, we investigated resting-state fMRI data along with extensive clinical, lifestyle, and genetic data collected from 37,842 UK Biobank participants, with the object of elucidating intersubject variability in the fluctuation amplitudes of RSNs. Functional properties of the RSN amplitudes were first examined by analyzing correlations with the well-established between-network functional connectivity. It was found that a network amplitude is highly correlated with the mean strength of the functional connectivity that the network has with the other networks. Intersubject clustering analysis showed the amplitudes are most strongly correlated with age, cardiovascular factors, body composition, blood cell counts, lung function, and sex, with some differences in the correlation strengths between sensory and cognitive RSNs. Genome-wide association studies (GWASs) of RSN amplitudes identified several significant genetic variants reported in previous GWASs for their implications in sleep duration. We provide insight into key factors determining RSN amplitudes and demonstrate that intersubject variability of the amplitudes primarily originates from differences in temporal synchrony between functionally linked brain regions, rather than differences in the magnitude of raw voxelwise BOLD signal changes. This finding additionally revealed intriguing differences between sensory and cognitive RSNs with respect to sex effects on temporal synchrony and provided evidence suggesting that synchronous coactivations of functionally linked brain regions, and magnitudes of BOLD signal changes, may be related to different genetic mechanisms. These results underscore that intersubject variability of the amplitudes in health and disease need to be interpreted largely as a measure of the sum of within-network temporal synchrony and amplitudes of BOLD signals, with a dominant contribution from the former.

## Highlights

• Variability in amplitude of resting-state networks (RSNs) was assessed across 37,842 subjects.
• Network amplitudes are closely linked to functional connectivity between RSNs.
• Temporal synchrony between brain regions is a key factor determining RSN amplitudes.
• Sex effects on temporal synchrony differ between sensory and cognitive RSNs.
• Genetic variants associated with RSN amplitudes overlap with those associated with synchrony.

## 1 Introduction

Functional magnetic resonance imaging (fMRI) is a neuroimaging technique to measure brain activity using blood oxygenation level dependent (BOLD) signals. In the past three decades, fMRI studies have improved our understanding of the role and function of each brain region, and how different brain regions are functionally interconnected. Among the various analysis methods (e.g., clustering-based (Bellec et al., 2010; Yeo et al., 2011) and graph-based (Power et al., 2011; Rubinov and Sporns, 2010) analyses), independent component analysis (ICA) has become one of the most popular tools to investigate brain networks comprising multiple spatially distributed, but functionally interacting, brain regions. ICA decomposes a resting-state fMRI dataset into multiple components; these distinct components are also referred to as “RSNs” (resting-state networks) or “nodes” (Smith et al., 2013), depending on the context. ICA-based studies have found that the architectures of large-scale neuronal communications during resting state are highly reproducible across different studies (Chen et al., 2008; Damoiseaux et al., 2006; Li et al., 2012; Meindl et al., 2010) and have high correspondence with those occurring across different task conditions (Nickerson, 2018; Smith et al., 2009). These results suggest that resting-state networks (RSNs) reflect the intrinsic functional organization of the brain. An increasing number of resting-state fMRI studies have explored the intersubject variability in these functional networks, to better understand how this can be related to unique traits of individuals, such as mental states (Geerligs et al., 2015) or personality (Nostro et al., 2018), as well as disease (Zhang and Raichle, 2010). Recent efforts to create large-scale neuroimaging databases such as the UK Biobank (UKB) are expected to foster new findings by leveraging fMRI data acquired from tens of thousands of people.

RSNs can be explored using different approaches. Functional connectivity (FC, i.e., temporal correlations) *between* RSNs has been extensively investigated, revealing that the FC profile is unique to individuals (Finn et al., 2015). In addition, intersubject variability in the connectivity patterns can predict cognitive behavior (Cabral et al., 2017; Lin et al., 2018; van den Heuvel and Hulshoff Pol, 2010) and disease state (Craddock et al., 2009; Dubbelink et al., 2014; Liu et al., 2008). Another popular way to investigate RSNs is to examine spatial maps that represent the FC of each voxel to a given network, using dual regression analysis (Beckmann et al., 2009; Nickerson et al., 2017). This makes it possible to identify differences in spatial locations or shapes of FC between participants, or a group of participants. Compared with the methods based on FC, the amplitudes of spontaneous fluctuations of RSNs, which can be obtained from the standard deviation of the RSNs’ timeseries, have been much less studied. The RSN timeseries have been primarily used to derive the aforementioned FC measurements (correlations between RSNs), and few studies have looked into the intersubject *variability* in the amplitudes of the RSN timeseries.

Applying data-driven multivariate analyses, a multimodal UK Biobank (UKB) brain imaging study on 5,430 participants revealed several “modes” of population covariation between image-based measures and non-image based measures (e.g., demographic, lifestyle), which included a covariation between fMRI measures (network amplitude and FC) and aging-related process (Miller et al., 2016). Two more recent studies focused more directly on network amplitude itself. One study investigated whether the amplitudes of RSNs are associated with brain states by assessing their performance on discriminating different brain states in the fMRI data collected during rest and task conditions (Sala-Llonch et al., 2019). It was found that the discrimination performance of the amplitudes of the RSNs derived from high-dimensional ICA (>100 components) was comparable to those obtained using between-network FC, capturing information relevant to different brain states. In Bijsterbosch et al. (2017), within-subject and intersubject variability in the amplitudes were investigated using resting-state fMRI scans acquired from two different public datasets, UKB and the Human Connectome Project (HCP). The results showed that the networks responsible for similar brain functions (e.g., processing visual information) have similar covariation of their amplitudes across participants. Analyzing the fMRI data collected from the HCP participants who were scanned twice on the same day, it was further shown that the intra-subject variability in the amplitudes was related to the sleep duration of the participants, which is known to alter vigilance and arousal states. To our knowledge, there have been only these three studies (Bijsterbosch et al., 2017; Miller et al., 2016; Sala-Llonch et al., 2019) reporting associations between the RSN fluctuation amplitudes and individuals’ behavioural traits. Therefore, the associations with broad individual traits (e.g., biophysical, lifestyle) remain largely unknown.

It is worth noting that the RSN amplitude investigated in this work, which we refer to as “network amplitude” in the remainder of the article, is different from the voxel-level metrics such as amplitude of low-frequency fluctuation (ALFF) or fractional ALFF (fALFF) (Zang et al., 2007; Zou et al., 2008). Indeed, in our study, the network amplitude is derived from the network-level timeseries obtained by weighted-averaging the timeseries of all voxels within the network, whereas, in ALFF/fALFF analysis, amplitudes are derived from the voxel-level timeseries. Therefore, network amplitude is a highly compact indicator that summarizes each RSN in one scalar value. Network amplitude has so far been understood in the context that it represents the magnitude of the BOLD activity in the RSN (Bijsterbosch et al., 2017; Nickerson et al., 2017), but it has not been studied in great detail. This motivated us to investigate the relationship between network amplitude derived from the network-level timeseries and BOLD amplitude defined as the average voxelwise BOLD fluctuation amplitudes within a network.

In this work, we comprehensively assessed network amplitudes to understand their variations across participants, and to identify the factors determining intersubject variability using UKB data collected from 37,842 participants. The dataset consists of the resting-state fMRI scans and a wide range of physical, health-related, sociodemographic, lifestyle, and genetic information on the individuals.

In the first part of this study, we show how intersubject variability in network amplitudes is related to differences in imaging, non-imaging, and genetic phenotypes:

- FC between RSNs was chosen as the imaging phenotype to associate with network amplitudes, as it is derived from the same RSN timeseries and intimately related to amplitude changes (Duff et al., 2018), yet measures a different property of the RSNs (Cole et al., 2016; Duff et al., 2018; Friston, 2011).
- We conducted clustering and correlation analyses to relate the network amplitudes to 4,897 non-imaging variables such as systolic blood pressure and year ended full time education.
- We carried out population-based genome-wide association studies (GWASs) to discover genetic variations associated with the network amplitudes.

In the second part of the study, the key factors contributing to the intersubject variability of the network amplitudes were investigated. We show that the primary factor is the temporal synchrony between the spontaneous fluctuations of the distributed brain regions involved in a given RSN, rather than the average voxelwise BOLD fluctuation amplitudes of the regions (mathematically, network amplitude should be largely driven by a combination of these two measures). This finding has significant implications in interpreting the downstream analysis results. We demonstrate that associations of network amplitudes with the FC between RSNs, non-imaging variables, and genetic phenotypes are mostly driven by the temporal synchrony, by conducting the following analyses:

- The correlations between network amplitudes and FC across participants were compared with the correlations between temporal synchrony and FC, and between BOLD amplitudes and FC.
- Linear regression analyses were conducted with non-imaging variables of interest as predictors and either temporal synchrony or BOLD amplitude as a dependent variable.
- GWASs of temporal synchrony and separate GWASs of BOLD amplitude were carried out to determine which set of associated genetic variants was more similar to the set identified from GWASs of network amplitude.

## 2 Material and methods

### 2.1 Imaging data

This study used imaging and non-imaging variables derived from the January 2020 UKB data release. This contains brain imaging datasets (T1, T2 FLAIR, susceptibility-weighted MRI, resting fMRI, task fMRI, and diffusion MRI) of 41,985 participants collected at three sites (Stockport, Newcastle, and Reading), all having identical imaging hardware (Alfaro-Almagro et al., 2021). The brain imaging data were acquired using a Siemens Skyra 3T scanner with a standard Siemens 32-channel RF receive head coil at each site. Before the resting-state functional scans, T1-weighted images of the entire brain were acquired for 5 min (repetition time = 2000 ms, echo time = 2.01 ms, flip angle = 8°, resolution = 1 × 1 × 1 mm, field of view = 208 × 256 × 256 matrix). For the resting-state functional scans, BOLD contrast echo-planar (EPI) T2*-weighted images (repetition time = 735 ms, echo time = 39 ms, flip angle = 52°, resolution = 2.4 × 2.4 × 2.4 mm, field of view = 88 × 88 × 64 matrix) were acquired for 6 min (490 timepoints). Participants were instructed to lie still, keeping their eyes fixated on a crosshair. The full acquisition protocol can be seen at http://biobank.ctsu.ox.ac.uk/crystal/refer.cgi?id=2367.

The datasets were processed with an automated processing and quality control (QC) pipeline (Alfaro-Almagro et al., 2018), and unusable datasets (e.g., because of incomplete data, or severe MRI artifacts) were removed. To briefly describe the preprocessing pipeline, the anonymized (“defaced”) T1 structural images were preprocessed with the following steps: gradient distortion correction using the tools developed by FreeSurfer and HCP teams (Glasser et al., 2013), removal of non-brain structure (e.g., neck) and brain extraction using FLIRT (FMRIB's Linear Image Registration Tool) (Jenkinson et al., 2002; Jenkinson and Smith, 2001) and BET (Brain Extraction Tool) (Smith, 2002), linear alignment to MNI152 space using FLIRT, and non-linear registration to 1-mm MNI152 space using FNIRT (FMRIB's Nonlinear Image Registration Tool) (Andersson et al., 2007a, 2007b). The rfMRI data was preprocessed using Melodic (Beckmann and Smith, 2004) that performs EPI unwarping, gradient distortion correction, motion correction, grand-mean intensity normalisation, and highpass temporal filtering, followed by FMRIB's ICA-based X-noiseifier (Beckmann and Smith, 2004; Griffanti et al., 2014; Salimi-Khorshidi et al., 2014) to remove structured artefacts. The preprocessed data were then aligned to T1 space using FLIRT and then transformed to the standard MNI152 space using the previously obtained nonlinear transformation from T1 space to the MNI space. All preprocessing steps are described in great detail in Alfaro-Almagro et al. (2018).

In this study, we used data from the 37,842 participants (17,721 males and 20,121 females; 64.1 ± 7.5 years old) with usable T1 and rfMRI data in the MNI space. We only used the first-scan data from the 1,503 out of the 37,842 participants with repeat-scan data.

### 2.2 Dual regression

**Fig. 1:**
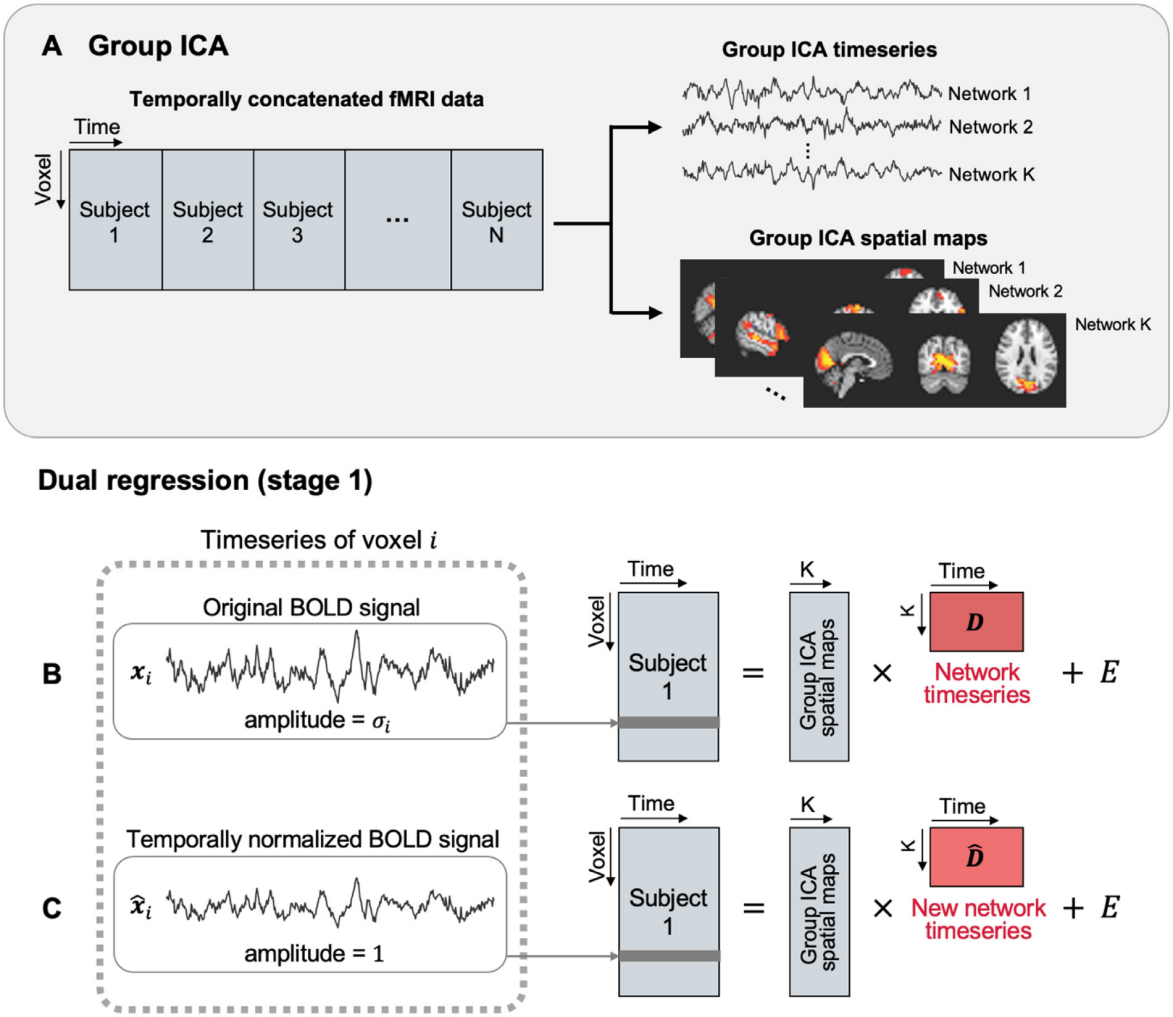
**(A)** Schematics of temporal concatenation group-ICA. Group-ICA decomposes the temporally concatenated fMRI data from participants into a set of independent spatial maps and a set of corresponding timeseries. The ICA dimension (*K*) was set to 25 in this study. The spatial maps from group ICA are used in the first stage of dual regression to derive subject-specific network timeseries, which are then subsequently used in the second stage of the dual regression to obtain subject-specific spatial maps. **(B)** Traditional dual regression stage 1. Network timeseries (*D*) are obtained using the original fMRI data, where the BOLD signal in each voxel ($x_{i}$) fluctuates with standard deviation $\sigma_{i}$. Network amplitudes are defined as the standard deviations of the estimated network timeseries. E denotes residuals. **(C)** New, distinct network timeseries ($\hat{D}$) are obtained using temporally normalized fMRI data, where the standard deviation of the BOLD signal is set to 1 for every voxel. Temporal synchrony of each network is defined as the standard deviation of each of these new network timeseries.

Group ICA with temporal concatenation was performed using FSL-MELODIC ICA (Beckmann et al., 2005) with 25 ICA dimensions (Miller et al., 2016). The ICA output is a collection of spatial maps of RSNs that are common to all participants and that closely match those frequently found in resting-state fMRI studies (Fig. 1A) (Damoiseaux et al., 2006; Smith et al., 2009).

The group spatial maps were then used in a dual regression analysis, to estimate subject-specific network timeseries and spatial maps (Fig. 1B); these can then be used to compare differences across participants (Beckmann et al., 2009; Nickerson et al., 2017).

Of the 25 group spatial maps, four components were non-neural components (e.g., motion artifacts), and their timeseries were discarded in further analyses. The maps of the 21 group ICA components can be viewed in Fig. S1 and at https://www.fmrib.ox.ac.uk/ukbiobank/group_means/rfMRI_ICA_d25_good_nodes.html.

### 2.3 Extracting subject-specific amplitudes and temporal synchrony

#### 2.3.1 Network amplitude

In stage 1 of dual regression, subject-specific network timeseries (D) are extracted as the following:

(1) X=GD+E

(2) $$D={\left(G^{T}G\right)}^{-1}G^{T}X$$

where $X={\left[x_{1},\;x_{2},\;\cdots ,\;x_{N}\right]}^{T}\;\in R^{N\times T}$ is the fMRI data matrix of a single participant, $x_{i}\in R^{T\times 1}$ (i=1,2,⋯,N) is the BOLD timeseries of voxel i, $G=[g_{1},\;g_{2},\;\cdots ,\;g_{K}]$ $\in R^{N\times K}$ is the K group spatial maps, $E\in R^{N\times T}$ is the residuals, $D={\left[d_{1},\;d_{2},\;\cdots ,\;d_{K}\right]}^{T}$ $\in R^{K\times T}$ is the matrix of K network timeseries, and N and T are the number of voxels and time points, respectively.

The amplitude of each network is defined as the standard deviation of its timeseries, $a_{k}=\sqrt{var(d_{k})}$. The term ${\left(G^{T}G\right)}^{-1}$ in Eq. (2) is typically close to being a diagonal matrix (because of the independence assumption between sources in ICA), and essentially plays a role to scale each row in the term, $G^{T}X\in R^{K\times T}$. As the group ICA spatial maps G are common across participants, the scaling effects of ${\left(G^{T}G\right)}^{-1}$ are common across all participants, and therefore they do not contribute to relative intersubject differences in the network amplitudes. Each row of $G^{T}X$ is a timeseries of network k (before multiplying ${\left(G^{T}G\right)}^{-1}$), and can be expressed as $g_{k}^{T}X\;\in R^{1\times T}$, where $g_{k}={\left[g_{k}\left(1\right),\;g_{k}\left(2\right),\;\cdots ,\;g_{k}\left(N\right)\right]}^{T}\in R^{N\times 1}$ is the vectorized k^th^ group spatial map. It can be further expressed as

(3) $$g_{k}^{T}X=g_{k}\left(1\right)\cdot x_{1}^{T}+g_{k}\left(2\right)\cdot x_{2}^{T}+\ldots +\;g_{k}\left(N\right)\cdot x_{N}^{T}$$

which is a weighted sum of the BOLD timeseries $x_{1},\;x_{2},\;\ldots ,\;x_{N}$, and the weights are the elements in the k^th^ group spatial map.

As the intersubject variability in network amplitudes is determined by the term $g_{k}^{T}X$, examination of Eq. (3) provides a clue to understanding two main factors contributing to the variability:

(1) The degree of temporal synchrony across the BOLD timeseries “within” the group spatial map $g_{k}$.
(2) The amplitudes of the BOLD voxels’ timeseries ($x_{1},\;x_{2},\;\ldots ,\;x_{N}$).

**Fig. 2:**
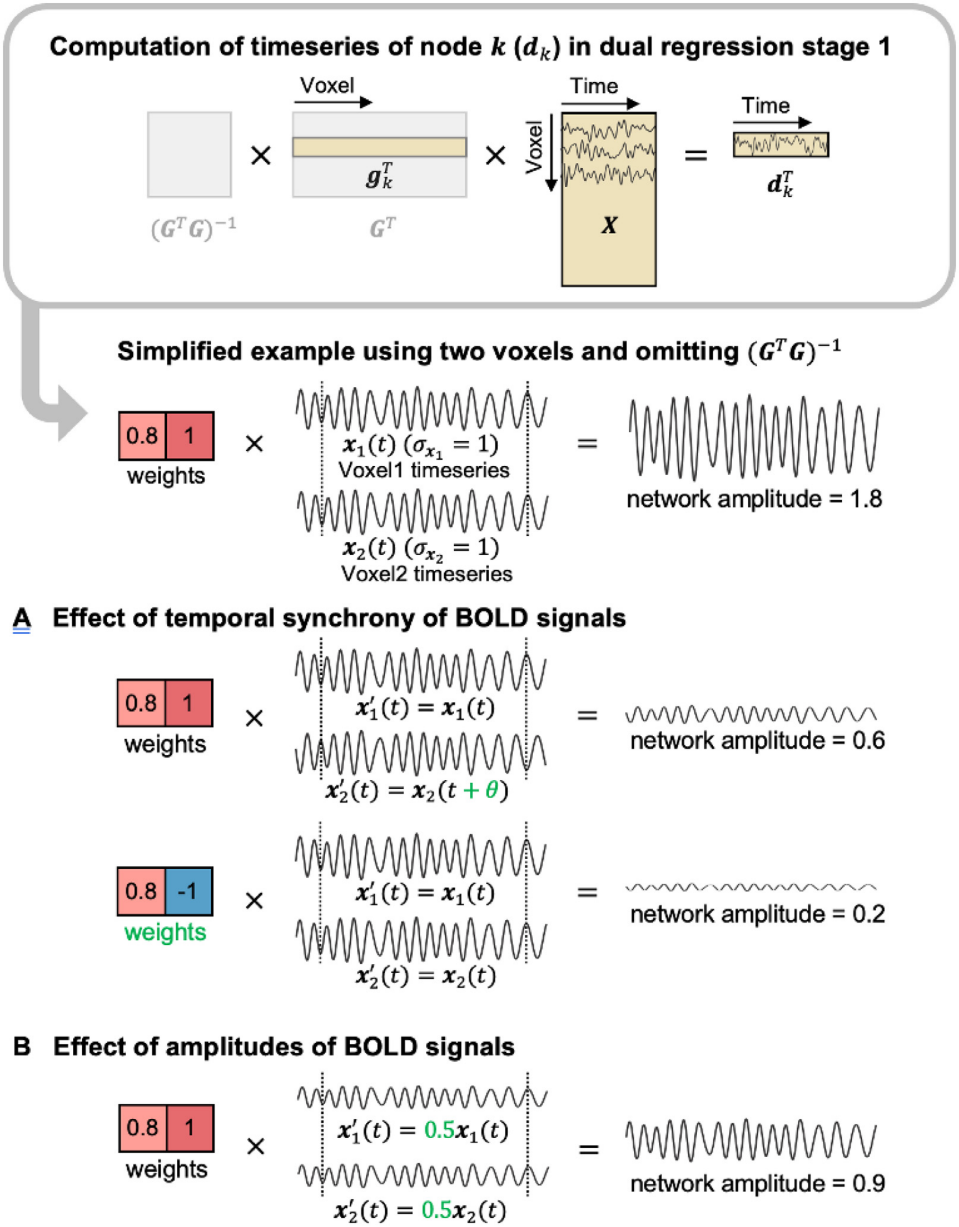
Effects of temporal synchrony and amplitudes of BOLD signals on network amplitude computed in dual regression Stage 1. The timeseries $d_{k}$ of network k corresponds to row k of ${\left(G^{T}G\right)}^{-1}G^{T}X$. ${\left(G^{T}G\right)}^{-1}$ does not affect intersubject differences in the network amplitude, and therefore is greyed out in the figure. A simplified example of $g_{k}^{T}X$ is presented below using two voxels. For illustration purpose, in the toy example, $x_{1}\left(t\right)$ and $x_{2}\left(t\right)$ are described as voxel timeseries of the same frequency that are perfectly aligned with a phase difference of 0. **(A)** Effect of temporal synchrony of BOLD signals. The network amplitude decreases due to the phase differences (θ) between the two timeseries denoted in green. The network amplitude is also small when the two voxels are expected to be anticorrelated based on the ICA weights (denoted in green) but their timeseries are positively correlated. **(B)** Effect of amplitudes of BOLD signals. As the BOLD signal amplitudes become half (denoted in green) – assuming the synchrony is unchanged – the network amplitude decreases by half.

Fig. 2 describes how these two factors affect the network amplitude. The second factor is apparent (Fig. 2B): it is easy to conjecture that high BOLD fluctuation amplitudes would give rise to high network amplitudes. Although it is not clear at first glance, it can be seen from Fig. 2A that high BOLD fluctuations alone cannot generate high network amplitudes: even if the amplitudes of BOLD fluctuations were to double, the final amplitude of the RSN timeseries could be small if the two fluctuations are not temporally synchronised, or they are temporally synchronised when they are expected to be anticorrelated according to the group spatial map. In short, network amplitude becomes larger when the BOLD fluctuations of the brain regions involved in the network are temporally synchronised and the BOLD fluctuation amplitudes themselves are high. Below, we attempt to dissociate these two factors by separately estimating and investigating “temporal synchrony” and BOLD amplitudes.

#### 2.3.2 Temporal synchrony

Let $\hat{X}={\left[{\hat{x}}_{1},\;{\hat{x}}_{2},\;\cdots ,\;{\hat{x}}_{N}\right]}^{T}\in R^{N\times T}$ be a matrix of temporally normalized fMRI data (Fig. 1C). The temporally normalized BOLD timeseries of voxel i is computed as ${\hat{x}}_{i}=z_{i}$, where $z_{i}=\left(x_{i}-\mu_{i}\right)/\sigma_{i}$ and $\mu_{i}$ and $\sigma_{i}$ are the mean and standard deviation of $x_{i}$. Similar to Eq. (2), $\hat{D}$ are computed using the spatial regression (dual regression stage 1):

(4) $$\hat{X}=G\hat{D}+E$$

(5) $$\hat{D}={\left(G^{T}G\right)}^{-1}G^{T}\hat{X}$$

Temporal synchrony is defined as the standard deviation of the timeseries in $\hat{D}$: ${\hat{a}}_{k}=\sqrt{\mathrm{var}({\hat{d}}_{k})}\;\left(k=1,\;\cdots ,\;K\right)$. Comparing Eq. (5) with Eq. (2), the term ${\left(G^{T}G\right)}^{-1}$ remains the same, and $G^{T}X$ is replaced with $G^{T}\hat{X}$ in which each row is a weighted sum of the normalized BOLD timeseries ${\hat{x}}_{1},\;{\hat{x}}_{2},\;\cdots ,\;{\hat{x}}_{N}$ that have variance equal to one. Therefore, the temporal synchrony is not influenced by the variance of the BOLD timeseries, but only affected by their temporal covariations (synchronisation with each other) and the ICA weights. In other words, the temporal synchrony ${\hat{a}}_{k}$ will be large when the voxels contributing strongly to the group spatial map ($g_{k}$) fluctuate in a synchronised way over time.

#### 2.3.3 BOLD amplitude

A BOLD amplitude is defined for each network to represent the average BOLD fluctuation amplitudes of the voxels that contribute strongly to the network. A threshold of |Z| = 3.29 ($P\left(|Z|\;>\;3.29\right)=10^{-3}$) was used to create binary masks $M=\left[m_{1},\;m_{2},\;\cdots ,\;m_{K}\right]\in R^{N\times K}$ to select the voxels for each network, where $m_{k}\in R^{N\times 1}$ is a binary mask of network k. The elements of $m_{k}$ are

(7) $$\begin{array}{c} m_{k}\left(i\right)=1,\;\mathrm{if}\;\left|g_{k}\left(i\right)\right|>3.29\\ m_{k}\left(i\right)=0,\;\mathrm{otherwise}. \end{array}$$

The BOLD amplitude of network k is then defined as ${\dot{a}}_{k}=\frac{1}{\sum_{i=1}^{N}m_{k}\left(i\right)}m_{k}^{T}v$ where $v={\left[\sqrt{\mathrm{var}(x_{1})},\;\cdots ,\;\sqrt{\mathrm{var}(x_{N})}\right]}^{T}\in R^{N\times 1}$ is a vector of the BOLD fluctuation amplitudes of N voxels.

The BOLD amplitude defined here is similar to the average of ALFFs over the thresholded regions in a network. Instead of using the binary mask $m_{k}$, we also tried two different weighted-averaging methods to obtain BOLD amplitudes (sum of standard deviation: ${\dot{a}}_{k}=\frac{1}{N}g_{k}^{T}v$; root sum of variance: ${\dot{a}}_{k}=\sqrt{{\left(g_{k}\circ g_{k}\right)}^{T}\left(v\circ v\right)}$) and found that they gave similar results (Fig. S3A).

### 2.4 Between-network FC

**Fig. 3:**
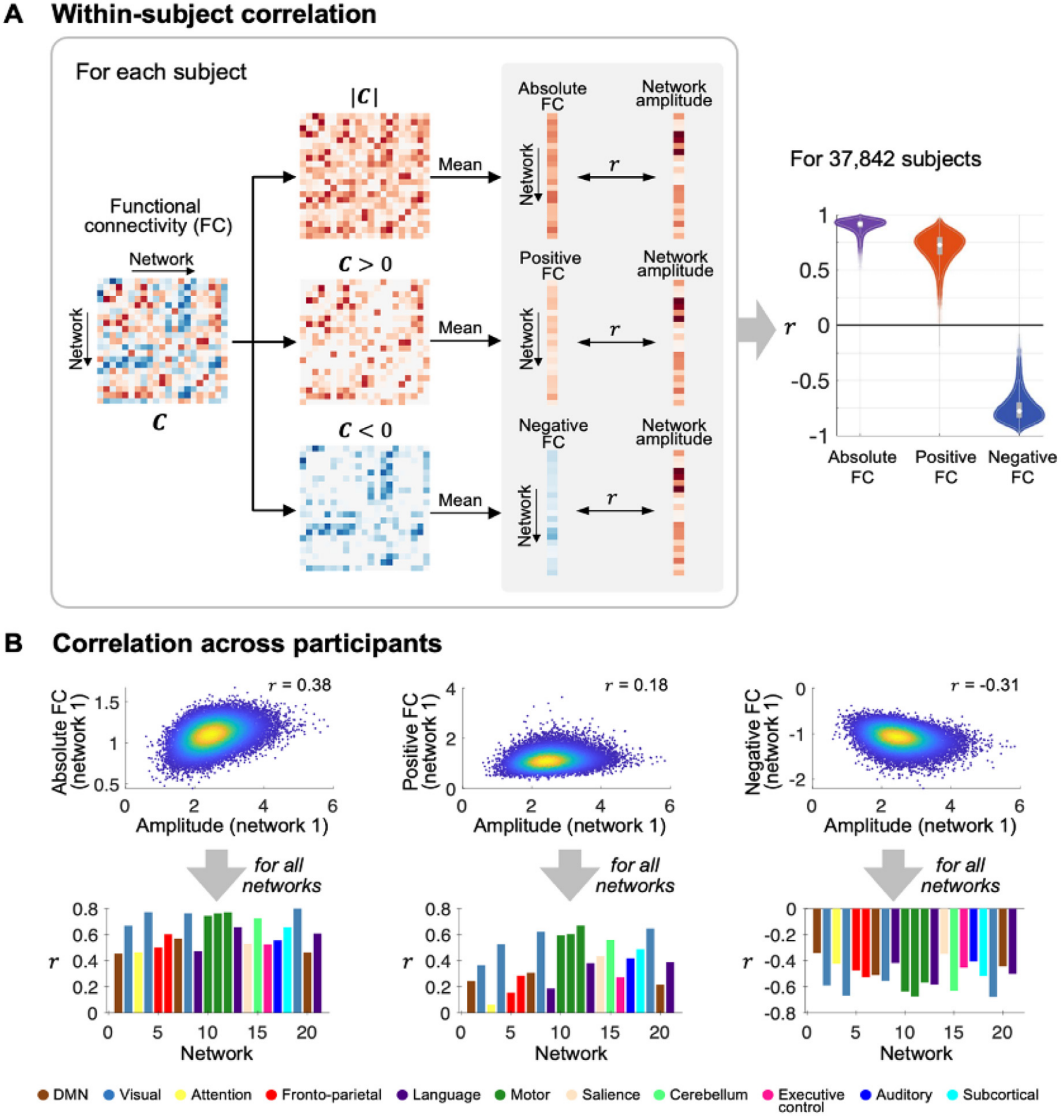
**(A)** Within-subject correlation. For each participant, the FC matrix is computed from network timeseries using partial correlation. Absolute values of the FC matrix are taken and averaged (across all other networks) for each network to compute the absolute FC. Similarly, positive and negative FC are computed using positive and negative elements in C, respectively. A Pearson correlation coefficient, r, is computed between network amplitudes and each of the absolute, positive, negative FC. The violin plots on the right-hand side shows the distributions of the correlation coefficients computed for 37,842 participants. **(B)** Correlation across participants. Top: illustrative scatter plots for network 1 (default mode network) amplitudes and each of the absolute, positive, and negative FC of network 1 computed from 37,842 participants are shown along with their correlation coefficients. Bottom: the bar graphs show the correlations obtained for all 21 networks.

The FC matrix $C=\left[c_{1},\;c_{2},\;\cdots ,\;c_{K}\right]\in R^{21\times 21}$ was computed by calculating partial correlation coefficients between the network timeseries. It was then subsequently used to calculate three types of summary connectivity strength for each network (Fig. 3), namely absolute, positive, and negative FC defined as below:

For each network k,

• Absolute FC: mean of $|c_{k}$|
• Positive FC: mean of positive FC in $c_{k}$
• Negative FC: mean of negative FC in $c_{k}$

(The diagonal elements in C were excluded from these calculations).

### 2.5 Clustering analysis of network amplitude

A hierarchical clustering analysis was performed to transform the network amplitude data into a low-dimensional space, such that the low-dimensional representation retains meaningful properties of the original data for further analyses.

A correlation matrix ($\in R^{21\times 21}$) was computed from the network amplitude matrix ($\in R^{\text{participant}\times 21}$), and Ward's clustering was performed on the correlation matrix using FSLNets (https://fsl.fmrib.ox.ac.uk/fsl/fslwiki/FSLNets), to discover a hierarchical structure of the similarity of the networks based on the intersubject covariation matrix. Based on the clustering result (which found one “sensory” cluster, and one “cognitive” one, similar to the clusters found in Bijsterbosch et al. (2017), we derived sensory amplitude $\in R^{\text{participant}\times 1}$ and cognitive amplitude $\in R^{\text{participant}\times 1}$ by taking the mean of the network amplitudes in each cluster.

### 2.6 Non-imaging variables

UKB data include a wide range of non-imaging variables covering demographic, health, and lifestyle information on the participants (https://www.ukbiobank.ac.uk/). In this work, a subset of non-imaging variables was selected using the following criteria to investigate their associations with network amplitudes:

• For cancer illness, non-cancer illness, treatment/medication, operative procedures, diagnoses variables: variables were selected if the number of the participants having a given condition was greater than 1% of the total participants included in our study.
• Other non-imaging variables: variables were selected if the number of non-missing records was greater than 50% of the total participants included in the study. Missing values were imputed based on soft-shrinkage SVD data reconstruction (nets_impute.m in FSLNets). The imputed data were used in the multiple linear regression analysis (Section 2.11), and the original data before imputation were used elsewhere.

With these criteria, a total of 4897 non-imaging variables were considered in this study. All the non-imaging variable names were kept the same as those used in the UKB showcase (https://biobank.ndph.ox.ac.uk/showcase/) to make it easier to identify them in the showcase for further information.

### 2.7 Confounding variables

For the UKB data, a recent study identified potential confounding factors to be considered when assessing associations between imaging and non-imaging variables (Alfaro-Almagro et al., 2021). Out of the full set of 602 confounding variables reported, we chose the optimal set of 184 confounds as proposed in (Alfaro-Almagro et al., 2018), consisting of 36 variables related to head motion to compensate for motion-related artifacts, 30 variables related to head size and scan position (to account for differences in the positions of the head and RF coil relative to the scanner), and 118 variables related to acquisition site and acquisition date/time, that can be important confounds in a multi-site study. Age and sex were not included in this set of confounding variables, as they were variables of interest in this study. The complete list of the selected confounds is provided in Table S1.

The confound-adjusted imaging and non-imaging variables were obtained via a regression-based unconfounding procedure (Alfaro-Almagro et al., 2021).

### 2.8 Genome-wide associations studies

Following the approach used in (Elliott et al., 2018; Smith et al., 2021), a genome-wide association study (GWAS) was carried out for these three measures for each network: network amplitude, temporal synchrony, and BOLD amplitude.

The second UKB release of imputed genetic data comprising over 90 million structural variants was used in this work. We estimated genetic effects with respect to the number of copies of the non-reference allele. Variants with minor allele frequency (MAF) below 1% or an imputation information score below 0.3 were first eliminated. Then, we used a maximal subset of unrelated participants with recent British ancestry determined using the variable *in.white.British.ancestry,* to minimize confounding effects of population structure and relatedness on the GWAS.

This QC filtering resulted in a total of 20,381,043 single-nucleotide polymorphisms (SNPs) and 33,287 participants (samples), which we partitioned at random into 22,172 participant discovery samples and 11,115 participant replication samples. GWAS was carried out using BGENIE v1.2 (https://jmarchini.org/bgenie/).

After running GWAS, the genetic variants significantly associated with the three different measures were determined using the standard GWAS P-value threshold of $-\log_{10}P=7.5$ (Elliott et al., 2018) in the discovery sample results.

To better understand the genetic effects of the identified genetic variants on the observed phenotypic variations, we referred to the genotype-tissue expression (GTEx) database (GTEx Consortium, 2017) that provides catalogs of expression quantitative trait loci (eQTLs) in 44 human tissues that have been made publicly available.

### 2.9 Relationships between network amplitude and individual traits

To establish relationships between network amplitude and non-imaging variables, we computed Pearson correlation coefficients between the sensory or cognitive amplitude and each of the 4897 non-imaging variables. The significance level adjusted for multiple testing across non-imaging variables using Bonferroni correction is $0.05/4,897=1.02\;\times 10^{-5}$. We used, however a more stringent threshold of $P=10^{-20},$ to determine significant correlations to ensure at least a modest strength of association (approximately corresponding to |r|>0.05). To find associations independent of age, we also computed the Pearson correlation coefficients between the sensory or cognitive amplitude and non-imaging variables after regressing out age from the amplitudes and variables. Statistical tests (Pearson and Filon, 1898)) for the comparison between two correlations were done using the R package ‘cocor’ (Diedenhofen and Musch, 2015).

### 2.10 Comparisons of amplitudes

Correlations between the network amplitudes, temporal synchrony, and BOLD amplitudes (see Section 2.3) across participants were computed for every network, to investigate how similarly they covary across participants.

Further, to investigate which voxels drive the similarity (if present) of the intersubject variations between the network amplitudes and temporal synchrony, we set a threshold value for the group ICA maps, to determine a subset of the voxels to include, when computing the temporal synchrony. By varying the threshold value, we computed (1) the temporal correlation between the network timeseries ($\hat{D}$ in Fig. 1C) and the one computed from the thresholded voxels, (2) the correlation between the original temporal synchrony and the one obtained from the thresholded voxels across participants, and (3) the correlation between the original network amplitudes and temporal synchrony obtained from the thresholded voxels across participants.

### 2.11 Multiple linear regression analysis

Relationships between non-imaging variables and each of the network amplitudes, temporal synchrony, and BOLD amplitudes were investigated using multiple linear regression analyses. We first selected a small subset of non-imaging variables from Table 3 that are most strongly correlated with sensory and/or cognitive amplitudes: systolic blood pressure, body fat %, haemoglobin concentration, and sleep duration. Note that we selected a representative variable from each category that is commonly used in the literature (e.g., systolic blood pressure from cardiovascular variables, body fat % from body composition variables). Age and sex (coded as 0 and 1 for female and male, respectively) were included as variables of interest along with age^2^ and interaction terms between age and sex (age × sex and age^2^ × sex) to model age and sex effects more comprehensively. A multiple linear regression analysis was performed with the non-imaging variables as independent variables and an imaging variable as the dependent variable. A total of 63 (= 21 networks × network amplitude/temporal synchrony/BOLD amplitude) multiple linear regression analyses were performed. All the dependent and independent variables (except for the sex variable) were normalised before fitting the model.

## 3 Results

### 3.1 Intersubject variability of network amplitude

We first focus on associations between the network amplitudes and phenotypes from three different domains: (1) FC, (2) non-imaging variables, and (3) genetics.

#### 3.1.1 Associations between network amplitude and FC

Fig. 3A shows the distribution of the within-subject correlations between the network amplitudes and absolute/positive/negative FC across 21 networks. For most of the participants, the network amplitudes and absolute FC were strongly correlated (r = 0.91 ± 0.044; mean ± standard deviation (SD)), indicating that networks with greater amplitudes tend to have stronger FC with other networks. Similarly, the network amplitudes were strongly correlated with positive (r = 0.71 ± 0.12) and negative (r = -0.75 ± 0.11) FC.

This correlation between amplitude and FC across participants is presented for each network in Fig. 3B. The correlation between the amplitude and absolute FC was significant for every network (Bonferroni corrected P < 0.001; r = 0.57 ± 0.14), indicating that participants with larger network amplitudes tend to also show stronger FC. Strong correlations (r > 0.7) were observed for networks 19, 12, 11, 4, 8, and 10 (descending order of the correlation strength), which are the visual (4, 8, 19) and motor (10, 11, 12) networks (Beckmann et al., 2005; Damoiseaux et al., 2006; Lee et al., 2013; Veer et al., 2010). Similarly, the correlations were found to be significant for all 21 networks (Bonferroni corrected P < 0.001) when the positive (r = 0.36 ± 0.19) or negative (r = -0.48 ± 0.11) FC was used separately. In line with what we observed for the absolute FC, these correlations were consistently the strongest for the visual (4, 8, 19) and motor (10, 11) networks.

#### 3.1.2 Relevance of network amplitude to non-imaging variables

**Fig. 4:**
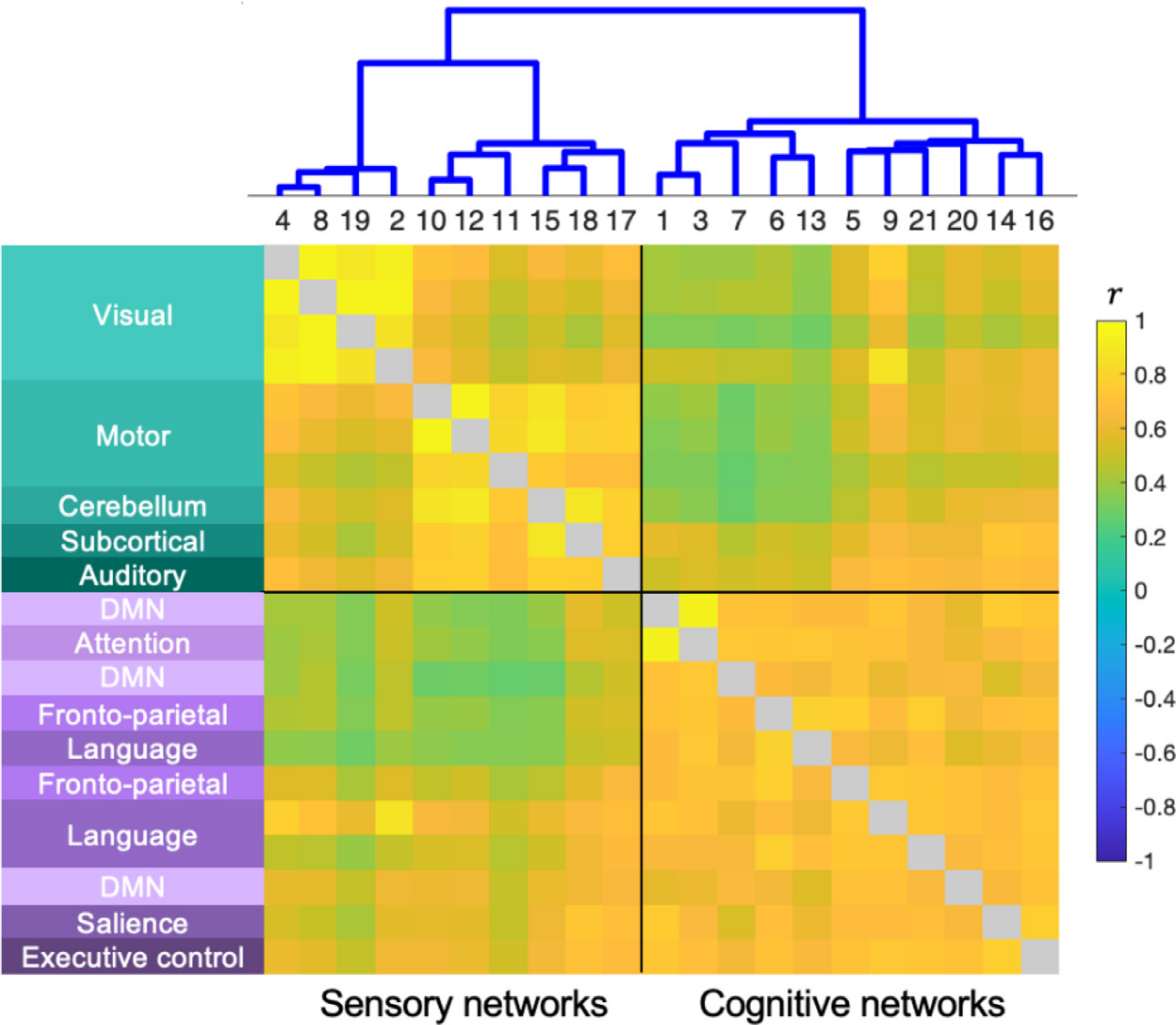
Ward's clustering shows a clear separation of the sensory networks (green) and cognitive networks (purple). The order of networks and dendrogram are presented on the top along with the correlation matrix ($\in R^{21\times 21}$) computed from the network amplitude matrix ($\in R^{\text{participant}\times 21}$). Each network is labeled with the conventional functional network name on the left (Beckmann et al., 2005; Damoiseaux et al., 2006; Lee et al., 2013; Veer et al., 2010).

The clustering result revealed two distinct groups of networks as shown in Fig. 4. The first group contained visual (2, 4, 8, 19), motor (10, 11, 12), cerebellum (15), auditory (17), and subcortical (18) networks, whereas the second group included the default mode (1), its more limbic (7) and precuneal component (20), the salience (14), attention (3), right and left fronto-parietal (5, 6), language-related (9, 13, 21), and executive control (16) networks. The hierarchical structure shows that the amplitudes of the networks with similar functions (e.g., visual) covary – i.e., vary across the participants in a similar manner – clearly separating the networks into two, mainly sensory and cognitive, clusters. In the remainder of this work, we refer to the first and second groups as “sensory” and “cognitive” groups based on the functional properties of the networks they contain.

Table 1 shows the top 50 non-imaging variables most significantly correlated with the sensory and cognitive amplitudes. Age was found to be most strongly correlated with both amplitudes. Cardiovascular factors, body composition, blood count, lung function (e.g., forced expiratory volume (FEV)), and sex-related variables (e.g., testosterone) were the next significant variables associated with sensory and cognitive amplitudes.

**Table 1:** List of top 50 non-imaging variables that are most significantly associated with each of the sensory and cognitive amplitudes (no age deconfounding). Unadjusted P values are displayed.

**Sensory amplitude**			**Cognitive amplitude**		
r	P	Non-imaging variable	r	P	Non-imaging variable
-0.244	< 1E-300	Age	-0.247	< 1E-300	Age
-0.208	1.1E-291	Cardiac index during PWA	0.230	1.2E-251	Year ended full time education
-0.197	7.7E-274	Cardiac output during PWA	-0.159	3.0E-183	Central systolic blood pressure during PWA
-0.185	7.8E-250	Systolic brachial blood pressure	-0.157	2.1E-180	Systolic brachial blood pressure
-0.183	2.5E-243	Central systolic blood pressure during PWA	-0.152	2.2E-167	Central pulse pressure during PWA
-0.179	3.9E-231	Peripheral pulse pressure during PWA	-0.151	9.9E-166	Peripheral pulse pressure during PWA
-0.179	6.8E-231	Central pulse pressure during PWA	-0.157	1.1E-165	Cardiac index during PWA
0.159	8.0E-203	Leg fat-free mass (right)	-0.150	7.8E-158	Cardiac output during PWA
0.157	9.8E-198	Arm fat-free mass (right)	-0.150	4.5E-157	Stroke volume during PWA
-0.153	2.1E-196	Current employment status	-0.127	1.4E-134	Current employment status
0.203	3.3E-195	Year ended full time education	-0.171	3.3E-131	Ever had breast cancer screening / mammogram
0.156	5.0E-193	Whole body water mass	-0.136	3.5E-126	Systolic blood pressure, automated reading
-0.163	2.4E-185	Stroke volume during PWA	0.117	5.7E-112	Father still alive
0.143	1.1E-170	Whole body fat-free mass	0.113	9.5E-106	Mother still alive
0.151	2.7E-169	Forced expiratory volume in 1 s (FEV1)	0.108	2.9E-97	Own or rent accommodation lived in
0.141	5.9E-167	Hand grip strength (left)	-0.107	5.0E-97	Touchscreen duration
0.140	3.7E-164	Hand grip strength (right)	-0.129	1.6E-93	Interpolated Age of participant when non-cancer illness first diagnosed
-0.155	3.9E-162	Systolic blood pressure, automated reading	-0.110	1.2E-88	Mean arterial pressure during PWA
0.146	2.0E-158	Forced vital capacity (FVC)	0.107	6.7E-88	Average total household income before tax
0.136	8.7E-155	Arm predicted mass (left)	0.110	1.1E-87	Total peripheral resistance during PWA
0.136	1.7E-154	Arm fat-free mass (left)	-0.103	4.9E-86	Mean carotid IMT (intima-medial thickness) at 150 degrees
-0.137	1.1E-149	Impedance of arm (left)	-0.108	1.9E-84	End systolic pressure during PWA
0.143	7.3E-147	Total peripheral resistance during PWA	-0.100	9.2E-84	Ever had bowel cancer screening
-0.135	3.0E-146	Impedance of arm (right)	0.102	2.2E-83	Haemoglobin concentration
0.124	7.7E-126	Mother still alive	0.099	5.1E-79	Haematocrit percentage
0.122	1.4E-123	Weekly usage of mobile phone in last 3 months	-0.096	2.5E-78	Length of time at current address
-0.130	5.6E-122	Mean arterial pressure during PWA	0.097	3.3E-76	Red blood cell (erythrocyte) count
0.137	1.1E-121	Forced expiratory volume in 1 s (FEV1), Best measure	0.100	2.3E-75	Forced expiratory volume in 1 s (FEV1)
0.120	3.7E-121	Own or rent accommodation lived in	-0.097	3.0E-75	Mean carotid IMT (intima-medial thickness) at 120 degrees
-0.128	3.1E-119	End systolic pressure during PWA	-0.096	1.9E-74	Minimum carotid IMT (intima-medial thickness) at 150 degrees
0.126	4.0E-115	Testosterone	-0.128	4.0E-74	Had menopause
-0.118	7.2E-115	Ever had bowel cancer screening	-0.096	4.4E-74	Maximum carotid IMT (intima-medial thickness) at 150 degrees
-0.158	1.2E-112	Ever had breast cancer screening / mammogram	0.094	4.0E-73	Weight
-0.116	3.2E-112	Work/job satisfaction	0.092	1.4E-72	Weight (pre-imaging)
0.120	6.0E-112	Peak expiratory flow (PEF)	-0.092	4.0E-71	Wears glasses or contact lenses
-0.116	1.3E-111	Leg fat percentage (right)	0.093	2.9E-70	Hand grip strength (left)
0.127	3.4E-105	Forced vital capacity (FVC), Best measure	0.092	1.3E-69	Hand grip strength (right)
0.113	2.2E-104	Seated height	-0.088	4.1E-65	Mean time to correctly identify matches
0.111	1.0E-102	Hands-free device/speakerphone use with mobile phone in last 3 month	-0.089	7.3E-65	Mean carotid IMT (intima-medial thickness) at 210 degrees
-0.110	9.8E-102	Impedance of whole body	0.088	8.9E-65	Length of mobile phone use
0.114	6.5E-99	Average total household income before tax	0.107	1.6E-64	Number of symbol digit matches attempted
0.110	1.4E-97	Red blood cell (erythrocyte) count	0.106	2.3E-63	Number of symbol digit matches made correctly
0.107	8.5E-97	Number in household	-0.093	8.0E-63	Central augmentation pressure during PWA
-0.107	4.0E-95	Body fat percentage	0.086	8.7E-63	Leg fat-free mass (right)
0.106	2.1E-94	Weight (pre-imaging)	-0.088	4.5E-62	Maximum carotid IMT (intima-medial thickness) at 120 degrees
0.109	5.1E-92	Body surface area	0.090	3.6E-61	Forced vital capacity (FVC)
-0.103	1.6E-89	Sleep duration	-0.087	7.0E-61	Minimum carotid IMT (intima-medial thickness) at 120 degrees
0.105	1.1E-88	Haemoglobin concentration	-0.087	1.7E-60	Duration to first press of snap-button in each round
-0.107	2.6E-88	Average heart rate	0.086	8.3E-60	Arm fat-free mass (right)
0.103	3.4E-87	Weight	0.083	3.3E-58	Hands-free device/speakerphone use with mobile phone in last 3 month

^a^Year the participants first finished full-time education (school, college or university). See https://biobank.ndph.ox.ac.uk/showcase/field.cgi?id=22501 for more detail.

**Fig. 5:**
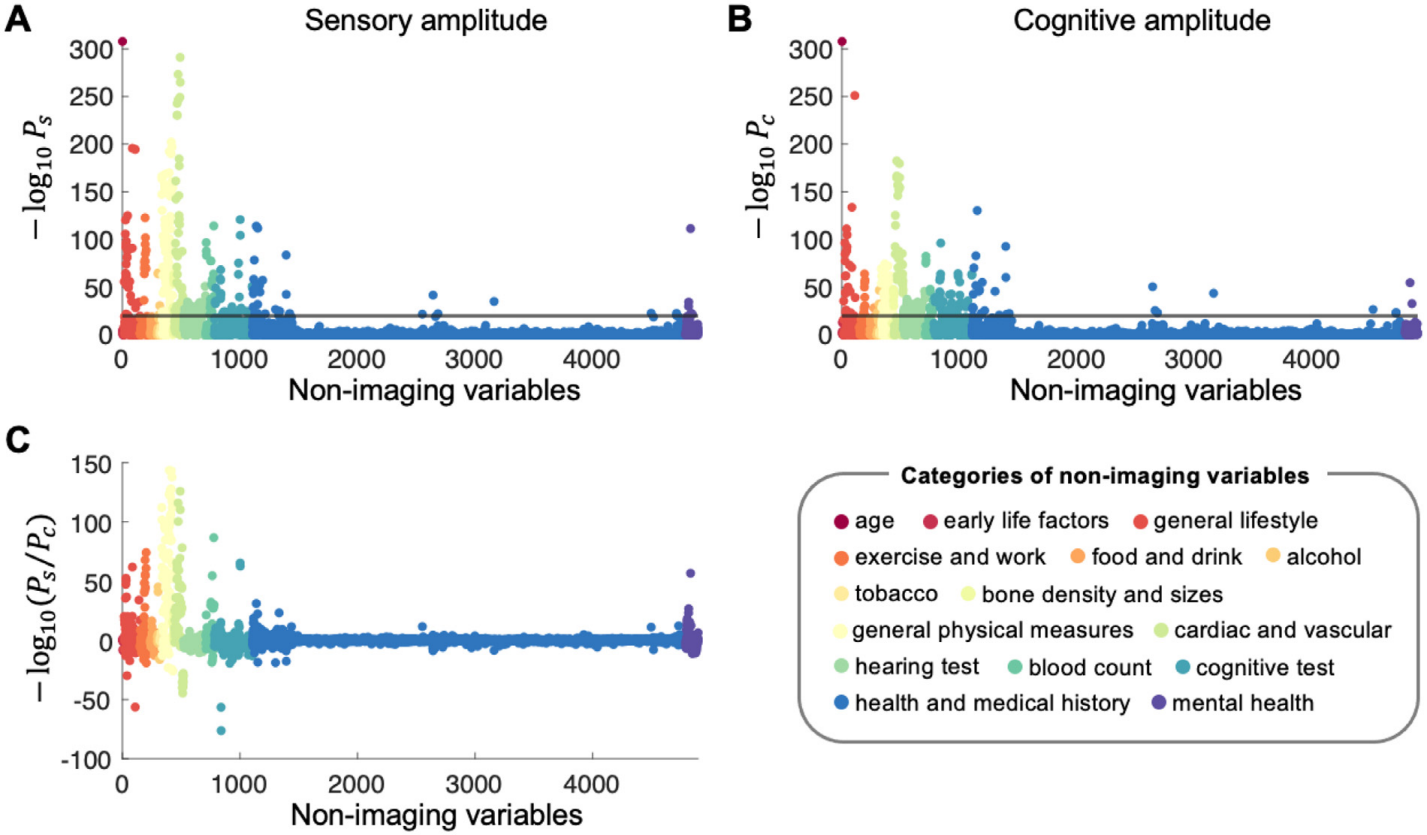
Manhattan plots showing the associations between 4,897 non-imaging variables and sensory/cognitive amplitude. The association strengths are presented as the Pearson correlation P values that have been converted to $-\log_{10}P$ (note: subscripts s and c indicate sensory and cognitive, respectively). The horizontal lines indicate $-\log_{10}P\;=20$. The non-imaging variables are categorized into 15 groups, which are denoted with different colours for visualization. **(A)** Associations between sensory amplitude and non-imaging variables. **(B)** Associations between cognitive amplitude and non-imaging variables. **(C)** Differences in the correlation P values between sensory and cognitive amplitudes. Variables with positive $-\log_{10}\left(P_{s}/P_{c}\right)$ have stronger associations with the sensory amplitude than cognitive amplitude.

To visualize the correlation profiles of the sensory and cognitive networks with the entire set of non-imaging variables, the P values were converted into $-\log_{10}P$ and displayed as Manhattan plots in Fig. 5A and B. The plots show that variables in the categories of age, cardiovascular measures, general physical measures, and blood count are most strongly correlated with both amplitudes. Differences in the correlation profiles between the sensory and cognitive networks are plotted in Fig. 5C, demonstrating that physical measures and cardiac and vascular variables are particularly more strongly associated with the sensory network than cognitive network. This trend was also observed when we examined the correlation profiles across each network (Fig. S2; Table S2).

To examine the differences in more detail, we investigated statistically detectable differences between cognitive and sensory amplitudes with respect to the magnitude of the correlations. In Table 2, we listed a total of 110 non-imaging variables that showed significantly different correlations between the sensory and cognitive amplitudes. Most of the cardiovascular and physical measures were more strongly correlated with the sensory amplitude, whereas 25 variables including year ended full time education, carotid artery thickness, touchscreen duration, and cognitive test performance showed higher correlations with the cognitive amplitude. There was no difference between the sensory and cognitive amplitudes in their correlation strengths with age.

**Table 2:** List of non-imaging variables with significant differences in their associations with the sensory and with the cognitive network amplitudes (no age deconfounding). Unadjusted P values are displayed. Variables more strongly associated with cognitive amplitude than sensory amplitude are denoted in bold.

r**(sensory)**	P**(sensory)**	r**(cognitive)**	P**(cognitive)**	$P_{\mathrm{diff}}$	Non-imaging variable
-0.208	1.1E-291	-0.157	1.1E-165	< 1E-300	Cardiac index during PWA
**0.203**	**3.3E-195**	**0.230**	**1.2E-251**	**1.1E-10**	**Year ended full time education**
-0.197	7.7E-274	-0.150	7.8E-158	< 1E-300	Cardiac output during PWA
-0.185	7.8E-250	-0.157	2.1E-180	6.7E-11	Systolic brachial blood pressure
-0.183	2.5E-243	-0.159	3.0E-183	1.2E-08	Central systolic blood pressure during PWA
-0.179	6.8E-231	-0.152	2.2E-167	4.4E-10	Central pulse pressure during PWA
-0.179	3.9E-231	-0.151	9.9E-166	1.2E-10	Peripheral pulse pressure during PWA
-0.153	2.1E-196	-0.127	1.4E-134	5.E-10	Current employment status
0.159	8.0E-203	0.086	1.2E-59	< 1E-300	Leg fat-free mass (right)
0.157	9.8E-198	0.086	8.3E-60	< 1E-300	Arm fat-free mass (right)
0.151	2.7E-169	0.100	2.3E-75	< 1E-300	Forced expiratory volume in 1 s (FEV1)
0.156	5.0E-193	0.078	1.8E-49	< 1E-300	Whole body water mass
0.143	7.3E-147	0.110	1.1E-87	1.8E-14	Total peripheral resistance during PWA
0.140	3.7E-164	0.086	2.2E-62	< 1E-300	Hand grip strength (right)
0.141	5.9E-167	0.080	9.9E-55	< 1E-300	Hand grip strength (left)
0.146	2.0E-158	0.090	3.6E-61	< 1E-300	Forced vital capacity (FVC)
0.143	1.1E-170	0.075	2.0E-47	< 1E-300	Whole body fat-free mass
-0.130	5.6E-122	-0.110	1.2E-88	5.9E-06	Mean arterial pressure during PWA
0.120	3.7E-121	0.101	1.3E-85	5.6E-06	Own or rent accommodation lived in
-0.128	3.1E-119	-0.108	1.9E-84	1.5E-06	End systolic pressure during PWA
0.136	8.7E-155	0.071	2.0E-42	< 1E-300	Arm predicted mass (left)
0.136	1.7E-154	0.070	2.9E-42	< 1E-300	Arm fat-free mass (left)
0.122	1.4E-123	0.081	1.0E-55	< 1E-300	Weekly usage of mobile phone in last 3 months
0.137	1.1E-121	0.093	2.4E-56	< 1E-300	Forced expiratory volume in 1 s (FEV1), Best measure
-0.137	1.1E-149	-0.051	2.2E-22	< 1E-300	Impedance of arm (left)
-0.135	3.0E-146	-0.053	4.4E-24	< 1E-300	Impedance of arm (right)
-0.116	3.2E-112	-0.081	1.5E-55	4.4E-16	Work/job satisfaction
0.111	1.0E-102	0.083	3.3E-58	7.4E-11	Hands-free device/speakerphone use with mobile phone in last 3 month
0.120	6.0E-112	0.070	6.9E-39	< 1E-300	Peak expiratory flow (PEF)
0.109	5.1E-92	0.085	3.9E-56	1.3E-08	Body surface area
0.127	3.4E-105	0.080	1.6E-42	< 1E-300	Forced vital capacity (FVC), Best measure
0.126	4.0E-115	0.061	2.2E-28	< 1E-300	Testosterone
0.107	8.5E-97	0.072	2.2E-44	2.2E-16	Number in household
0.113	2.2E-104	0.062	2.4E-32	< 1E-300	Seated height
**-0.071**	**2.1E-41**	**-0.103**	**4.9E-86**	**4.93E-14**	**Mean carotid IMT (intima-medial thickness) at 150 degrees**
0.098	3.1E-78	0.077	9.4E-49	9.5E-07	Creatinine (enzymatic) in urine
0.099	2.8E-80	0.074	2.2E-45	4.5E-09	Sitting height
0.098	2.5E-78	0.074	4.0E-46	5.8E-08	Sodium in urine
**-0.048**	**1.0E-20**	**-0.107**	**5.0E-97**	**< 1E-300**	**Touchscreen duration**
-0.116	1.3E-111	-0.022	2.8E-05	< 1E-300	Leg fat percentage (right)
-0.110	9.8E-102	-0.039	9.4E-14	< 1E-300	Impedance of whole body
**-0.065**	**3.0E-35**	**-0.097**	**3.0E-75**	**3.0E-13**	**Mean carotid IMT (intima-medial thickness) at 120 degrees**
**-0.066**	**2.2E-36**	**-0.096**	**4.4E-74**	**7.8E-12**	**Maximum carotid IMT (intima-medial thickness) at 150 degrees**
**-0.064**	**3.2E-34**	**-0.096**	**1.9E-74**	**1.6E-13**	**Minimum carotid IMT (intima-medial thickness) at 150 degrees**
-0.103	1.6E-89	-0.041	2.1E-15	< 1E-300	Sleep duration
0.098	1.8E-76	0.058	2.0E-28	< 1E-300	Drive faster than motorway speed limit
-0.095	9.3E-65	-0.071	2.4E-37	4.5E-08	SHBG
-0.107	4.0E-95	-0.020	1.3E-04	< 1E-300	Body fat percentage
0.094	4.0E-75	0.053	2.3E-24	< 1E-300	Number of vehicles in household
-0.107	2.6E-88	-0.034	4.1E-10	< 1E-300	Average heart rate
0.096	9.9E-60	0.075	9.9E-37	6.3E-07	Heel broadband ultrasound attenuation (left)
**-0.063**	**6.9E-33**	**-0.088**	**4.5E-62**	**1.1E-08**	**Maximum carotid IMT (intima-medial thickness) at 120 degrees**
0.091	4.3E-70	0.049	1.9E-21	< 1E-300	Time spent driving
**-0.055**	**6.1E-26**	**-0.089**	**7.3E-65**	**2.7E-15**	**Mean carotid IMT (intima-medial thickness) at 210 degrees**
0.103	1.4E-63	0.066	6.2E-27	< 1E-300	Length of working week for main job
0.101	1.2E-65	0.060	1.3E-24	< 1E-300	Average weekly beer plus cider intake
0.094	3.6E-72	0.044	3.4E-17	< 1E-300	Standing height
-0.104	2.7E-86	0.007	1.7E-01	< 1E-300	Leg fat percentage (left)
0.092	7.9E-55	0.069	2.0E-31	8.1E-08	Heel broadband ultrasound attenuation (right)
**-0.055**	**2.4E-25**	**-0.087**	**7.0E-61**	**1.3E-13**	**Minimum carotid IMT (intima-medial thickness) at 120 degrees**
0.089	3.9E-68	0.040	3.6E-15	< 1E-300	Height
**-0.051**	**2.4E-22**	**-0.084**	**2.3E-57**	**2.1E-14**	**Mean carotid IMT (intima-medial thickness) at 240 degrees**
-0.086	5.5E-54	-0.058	5.8E-25	2.7E-11	Apolipoprotein A
-0.099	6.0E-72	-0.026	2.2E-06	< 1E-300	Heart rate during PWA
**0.056**	**7.9E-27**	**0.078**	**5.7E-50**	**5.2E-07**	**Body mass index (BMI)**
**-0.052**	**9.3E-23**	**-0.081**	**2.1E-53**	**1.1E-11**	**Maximum carotid IMT (intima-medial thickness) at 210 degrees**
-0.084	1.8E-51	-0.057	1.8E-24	2.9E-10	HDL cholesterol
**-0.049**	**6.0E-21**	**-0.082**	**9.1E-55**	**3.9E-14**	**Minimum carotid IMT (intima-medial thickness) at 210 degrees**
-0.090	1.6E-68	-0.009	7.2E-02	< 1E-300	Arm fat percentage (right)
-0.093	3.0E-64	-0.020	2.6E-04	< 1E-300	Number of beats in waveform average for PWA
-0.089	6.2E-66	-0.006	2.5E-01	< 1E-300	Arm fat percentage (left)
**-0.064**	**1.0E-23**	**-0.087**	**4.5E-43**	**4.7E-08**	**Duration spent answering each puzzle**
**-0.046**	**2.6E-18**	**-0.077**	**2.9E-48**	**6.3E-13**	**Maximum carotid IMT (intima-medial thickness) at 240 degrees**
0.086	9.1E-59	0.019	3.5E-04	< 1E-300	Creatinine
-0.084	1.2E-59	-0.013	9.6E-03	< 1E-300	Trunk fat percentage
**-0.044**	**3.9E-17**	**-0.074**	**5.1E-45**	**3.9E-12**	**Minimum carotid IMT (intima-medial thickness) at 240 degrees**
0.080	1.3E-50	0.026	9.8E-07	< 1E-300	LV end systolic volume
-0.083	2.3E-51	-0.018	9.7E-04	< 1E-300	Ventricular rate
-0.066	3.7E-37	-0.044	3.3E-17	2.6E-07	Time spent watching television (TV)
-0.079	6.8E-53	0.003	5.7E-01	< 1E-300	Leg fat mass (right)
0.072	2.2E-41	0.036	1.9E-11	< 1E-300	Urate
**-0.055**	**4.2E-18**	**-0.078**	**1.5E-34**	**1.4E-07**	**Interval between previous point and current one in numeric path (trail #1)**
**0.054**	**1.9E-17**	**0.077**	**3.5E-33**	**2.0E-07**	**Amount of alcohol drunk on a typical drinking day**
0.078	9.4E-48	0.012	2.7E-02	< 1E-300	LV end diastolic volume
**0.054**	**2.4E-17**	**0.074**	**1.5E-31**	**2.3E-06**	**Number of puzzles correctly solved**
-0.072	6.0E-44	-0.016	2.2E-03	< 1E-300	Impedance of leg (right)
-0.086	7.6E-35	-0.049	3.9E-12	< 1E-300	QTC interval
-0.069	1.2E-40	-0.016	1.5E-03	< 1E-300	Impedance of leg (left)
0.065	2.5E-35	0.030	1.3E-08	2.2E-16	Risk taking
-0.062	6.8E-33	-0.033	1.5E-10	3.2E-11	Taking other prescription medications
0.079	3.8E-29	0.051	3.2E-13	1.6E-10	Heel Broadband ultrasound attenuation, direct entry
0.078	8.8E-29	0.051	2.9E-13	4.2E-10	Heel bone mineral density (BMD) T-score, automated
0.074	6.2E-26	0.048	6.2E-12	2.5E-09	Speed of sound through heel
0.086	1.8E-34	0.019	7.2E-03	< 1E-300	RR interval
0.064	2.2E-35	-0.006	2.2E-01	< 1E-300	Distance (Euclidean) to coast
-0.055	7.4E-25	-0.035	6.3E-11	3.6E-06	Cholesterol
0.082	2.4E-31	0.020	3.7E-03	< 1E-300	PP interval
**0.037**	**6.9E-09**	**0.065**	**6.2E-25**	**3.9E-11**	**Number of puzzles attempted**
**-0.029**	**3.5E-08**	**-0.055**	**6.7E-25**	**3.6E-09**	**Cystatin C**
0.058	4.7E-25	0.024	2.1E-05	3.3E-15	Pulse wave reflection index
-0.076	1.8E-27	-0.012	8.0E-02	< 1E-300	QRS num
0.059	2.1E-28	-0.002	7.0E-01	< 1E-300	LV stroke volume
**-0.024**	**4.6E-06**	**-0.051**	**3.1E-22**	**5.3E-10**	**Time spend outdoors in summer**
-0.061	1.3E-26	-0.007	2.1E-01	< 1E-300	Pulse rate, automated reading
**-0.003**	**6.2E-01**	**0.057**	**6.7E-27**	**< 1E-300**	**Trunk fat mass**
**-0.020**	**2.2E-04**	**-0.053**	**4.9E-23**	**2.2E-14**	**Urea**
0.053	2.8E-25	0.010	4.2E-02	< 1E-300	Operation code (1218 - vasectomy)
**-0.006**	**2.8E-01**	**0.055**	**9.6E-26**	**< 1E-300**	**Arm fat mass (right)**
0.053	4.2E-25	0.006	2.6E-01	< 1E-300	Skin colour
-0.048	7.2E-21	0.011	3.6E-02	< 1E-300	Whole body fat mass

To assess correlations independent of age, we computed the Pearson correlation coefficients after regressing out age from the amplitudes and the non-imaging variables. Table 3 shows that the same measures are still most strongly associated with the sensory and cognitive amplitudes independent of age. A total of 92 variables were correlated with the sensory and cognitive amplitudes with significantly different strengths independent of age (Table 4). Similar to the results in Table 2, most of the variables in Table 4 were cardiovascular measures, physical measures, blood count, and lung function, and they showed higher correlations with the sensory amplitude. Only three variables, year ended full time education (which was fairly highly correlated with age ended education, r = 0.470, P = 1.0E-300), touchscreen duration, and left ventricular (LV) stroke volume showed stronger correlations with the cognitive amplitudes than with the sensory amplitudes independent of age (Table 4).

**Table 3:** List of top 50 non-imaging variables that are most strongly associated with each of the sensory and cognitive amplitudes (after age deconfounding). Unadjusted P values are displayed.

**Sensory amplitude**			**Cognitive amplitude**		
r	P	Non-imaging variable	r	P	Non-imaging variable
-0.161	1.8E-173	Cardiac index during PWA	0.117	7.2E-111	Haemoglobin concentration
-0.156	6.7E-170	Cardiac output during PWA	0.116	1.0E-107	Haematocrit percentage
-0.141	4.6E-146	Systolic brachial blood pressure	-0.111	5.0E-91	Systolic brachial blood pressure
-0.131	1.5E-142	Impedance of arm (right)	-0.109	5.2E-87	Central systolic blood pressure during PWA
-0.130	1.4E-141	Impedance of arm (left)	0.099	1.1E-79	Red blood cell (erythrocyte) count
-0.136	6.6E-134	Central systolic blood pressure during PWA	-0.105	7.2E-78	Cardiac output during PWA
-0.131	2.5E-123	Peripheral pulse pressure during PWA	-0.106	2.1E-76	Cardiac index during PWA
0.130	6.1E-123	Testosterone	-0.100	1.4E-73	Peripheral pulse pressure during PWA
0.121	1.6E-121	Arm fat-free mass (right)	-0.099	1.3E-69	Stroke volume during PWA
0.121	4.3E-121	Arm predicted mass (left)	-0.097	1.2E-68	Central pulse pressure during PWA
0.120	2.9E-120	Arm fat-free mass (left)	-0.086	5.6E-54	Mean arterial pressure during PWA
-0.121	3.6E-117	Leg fat percentage (left)	-0.083	7.4E-54	Cardiac output
-0.119	3.8E-117	Impedance of whole body	-0.082	8.0E-47	Systolic blood pressure, automated reading
0.121	9.2E-117	Haemoglobin concentration	-0.074	7.6E-41	End systolic pressure during PWA
-0.121	2.3E-116	Leg fat percentage (right)	-0.070	1.6E-40	Volume of serum held by UKB
-0.126	1.0E-115	Central pulse pressure during PWA	0.074	2.5E-40	Total peripheral resistance during PWA
0.117	1.9E-114	Whole body fat-free mass	-0.063	2.4E-33	Volume of Li-Hep plasma held by UKB
0.119	1.7E-113	Haematocrit percentage	0.061	5.2E-32	Creatinine (enzymatic) in urine
0.117	1.2E-109	Whole body water mass	0.079	1.2E-30	Year ended full time education
0.114	1.1E-108	Leg fat-free mass (right)	0.064	1.3E-30	Testosterone
0.113	1.6E-107	Hand grip strength (left)	-0.058	1.5E-29	Touchscreen duration
0.112	2.3E-106	Hand grip strength (right)	-0.057	1.0E-28	Impedance of arm (right)
-0.115	5.3E-105	Body fat percentage	0.056	2.3E-27	Hand grip strength (right)
0.113	5.0E-102	Red blood cell (erythrocyte) count	-0.058	3.0E-27	LV stroke volume
-0.108	9.4E-93	Arm fat percentage (left)	-0.056	5.1E-27	Impedance of arm (left)
-0.114	2.8E-91	Stroke volume during PWA	0.063	1.1E-26	Heel quantitative ultrasound index (QUI), direct entry (left)
-0.104	3.9E-91	Arm fat percentage (right)	0.063	1.1E-26	Speed of sound through heel (left)
-0.108	1.9E-89	Average heart rate	0.056	3.6E-25	Average total household income before tax
-0.103	8.9E-86	Leg fat mass (right)	-0.053	1.2E-24	Treatment/medication code (1140879802 - amlodipine)
0.109	1.5E-85	Total peripheral resistance during PWA	0.054	2.1E-24	Urate
0.103	2.0E-84	Creatinine	0.052	4.4E-24	Arm predicted mass (left)
-0.106	4.4E-82	Mean arterial pressure during PWA	0.053	5.6E-24	Hand grip strength (left)
-0.103	3.1E-78	Heart rate during PWA	0.052	1.0E-23	Arm fat-free mass (left)
-0.102	1.1E-71	Systolic blood pressure, automated reading	0.052	1.3E-23	Sodium in urine
-0.097	6.5E-69	Number of beats in waveform average for PWA	0.058	3.6E-23	Speed of sound through heel (right)
-0.096	1.1E-67	End systolic pressure during PWA	0.058	6.6E-23	Heel quantitative ultrasound index (QUI), direct entry (right)
-0.089	2.5E-66	Sleep duration	0.051	8.1E-23	Arm fat-free mass (right)
0.091	6.4E-66	Urate	0.058	9.5E-23	Heel broadband ultrasound attenuation (left)
0.090	6.0E-61	Forced vital capacity (FVC)	-0.050	9.6E-23	Non-cancer illness code, self-reported (1065 - hypertension)
-0.085	8.8E-59	Trunk fat percentage	-0.054	6.5E-22	SHBG
0.083	1.5E-57	Creatinine (enzymatic) in urine	0.049	2.6E-21	Waist circumference
0.085	2.3E-57	Peak expiratory flow (PEF)	-0.047	9.8E-20	Vascular/heart problems diagnosed by doctor (4 - High blood pressure)
0.087	7.5E-57	Forced expiratory volume in 1 s (FEV1)	-0.049	1.1E-19	LV ejection fraction
-0.083	1.4E-51	Ventricular rate	-0.048	1.1E-19	Volume of EDTA2 plasma held by UKB
-0.076	3.7E-49	Impedance of leg (right)	0.047	1.9E-19	Weight
0.077	5.6E-49	Sodium in urine	-0.047	2.3E-19	Volume of EDTA1 plasma held by UKB
0.076	3.8E-48	Seated height	-0.046	4.7E-19	Diagnoses - secondary ICD10 (I10 - I10 Essential (primary) hypertension)
-0.074	3.1E-46	Impedance of leg (left)	0.046	6.3E-19	Weight (pre-imaging)
0.073	9.8E-45	Hands-free device/speakerphone use with mobile phone in last 3 month	0.046	1.0E-18	Whole body fat-free mass
-0.078	2.0E-44	SHBG	0.046	1.3E-18	Leg fat-free mass (right)

**Table 4:** List of non-imaging variables with significant differences in the associations with the sensory and cognitive amplitudes after regressing out age (after age deconfounding). Unadjusted P values are displayed. Variables more strongly associated with cognitive amplitude than sensory amplitude are denoted in bold.

r**(sensory)**	P**(sensory)**	r**(cognitive)**	P**(cognitive)**	$P_{\mathrm{diff}}$	Non-imaging variable
-0.161	1.8E-173	-0.106	2.1E-76	< 1E-300	Cardiac index during PWA
-0.156	6.7E-170	-0.105	7.2E-78	< 1E-300	Cardiac output during PWA
-0.141	4.6E-146	-0.111	5.0E-91	2.0E-12	Systolic brachial blood pressure
-0.136	6.6E-134	-0.109	5.2E-87	4.5E-10	Central systolic blood pressure during PWA
-0.131	2.5E-123	-0.100	1.4E-73	2.0E-12	Peripheral pulse pressure during PWA
-0.126	1.0E-115	-0.097	1.2E-68	5.9E-12	Central pulse pressure during PWA
-0.131	1.5E-142	-0.057	1.0E-28	< 1E-300	Impedance of arm (right)
-0.130	1.4E-141	-0.056	5.1E-27	< 1E-300	Impedance of arm (left)
0.130	6.1E-123	0.064	1.3E-30	< 1E-300	Testosterone
0.121	4.3E-121	0.052	4.4E-24	< 1E-300	Arm predicted mass (left)
0.121	1.6E-121	0.051	8.1E-23	< 1E-300	Arm fat-free mass (right)
0.120	2.9E-120	0.052	1.0E-23	< 1E-300	Arm fat-free mass (left)
-0.106	4.4E-82	-0.086	5.6E-54	1.7E-06	Mean arterial pressure during PWA
-0.119	3.8E-117	-0.045	4.3E-18	< 1E-300	Impedance of whole body
0.112	2.3E-106	0.056	2.3E-27	< 1E-300	Hand grip strength (right)
0.117	1.9E-114	0.046	1.0E-18	< 1E-300	Whole body fat-free mass
0.113	1.6E-107	0.049	9.6E-22	< 1E-300	Hand grip strength (left)
0.114	1.1E-108	0.046	1.3E-18	< 1E-300	Leg fat-free mass (right)
0.109	1.5E-85	0.074	2.5E-40	4.4E-16	Total peripheral resistance during PWA
0.117	1.2E-109	0.035	1.9E-11	< 1E-300	Whole body water mass
-0.117	4.5E-114	-0.020	8.2E-05	< 1E-300	Leg fat percentage (right)
-0.121	3.6E-117	-0.007	0.18	< 1E-300	Leg fat percentage (left)
-0.102	1.1E-71	-0.082	8.0E-47	2.7E-06	Systolic blood pressure, automated reading
-0.096	1.1E-67	-0.074	7.6E-41	2.6E-07	End systolic pressure during PWA
-0.115	5.3E-105	-0.002	0.72	< 1E-300	Body fat percentage
-0.108	1.9E-89	-0.032	1.9E-09	< 1E-300	Average heart rate
-0.105	1.2E-91	-0.020	1.1E-04	< 1E-300	Arm fat percentage (left)
-0.104	3.9E-91	-0.021	3.8E-05	< 1E-300	Arm fat percentage (right)
0.103	2.0E-84	0.035	7.9E-11	< 1E-300	Creatinine
0.091	6.4E-66	0.054	2.1E-24	< 1E-300	Urate
0.083	1.5E-57	0.061	5.2E-32	3.4E-07	Creatinine (enzymatic) in urine
-0.103	8.9E-86	-0.004	0.48	< 1E-300	Leg fat mass (right)
-0.103	3.1E-78	-0.029	2.2E-07	< 1E-300	Heart rate during PWA
-0.097	6.5E-69	-0.022	8.5E-05	< 1E-300	Number of beats in waveform average for PWA
0.077	5.6E-49	0.052	1.3E-23	1.3E-08	Sodium in urine
-0.089	2.5E-66	-0.024	3.1E-06	< 1E-300	Sleep duration
0.090	6.0E-61	0.029	1.6E-07	< 1E-300	Forced vital capacity (FVC)
0.085	2.3E-57	0.032	1.3E-09	< 1E-300	Peak expiratory flow (PEF)
-0.078	2.1E-44	-0.054	6.5E-22	1.6E-08	SHBG
0.080	2.3E-42	0.058	9.5E-23	1.7E-07	Heel broadband ultrasound attenuation (left)
0.087	7.5E-57	0.030	3.6E-08	< 1E-300	Forced expiratory volume in 1 s (FEV1)
-0.085	8.9E-59	0.012	0.023	< 1E-300	Trunk fat percentage
0.076	6.1E-38	0.052	1.9E-18	2.0E-08	Heel broadband ultrasound attenuation (right)
-0.083	1.4E-51	-0.017	2.6E-04	< 1E-300	Ventricular rate
0.076	3.8E-48	0.022	2.2E-05	< 1E-300	Seated height
-0.076	3.7E-49	-0.019	3.1E-04	< 1E-300	Impedance of leg (right)
0.073	9.8E-45	0.029	1.3E-08	< 1E-300	Hands-free device/speakerphone use with mobile phone in last 3 month
0.064	4.3E-35	0.043	7.3E-17	1.6E-06	Length of mobile phone use
0.068	2.5E-36	0.042	4.8E-15	2.8E-09	Body surface area
0.081	3.5E-43	0.032	8.0E-08	< 1E-300	Forced expiratory volume in 1 s (FEV1), best measure
0.062	7.2E-34	0.043	5.5E-17	7.5E-06	Weight
-0.074	3.1E-46	-0.020	1.3E-04	< 1E-300	Impedance of leg (left)
0.079	1.0E-40	0.036	6.6E-10	< 1E-300	Average weekly beer plus cider intake
0.081	2.3E-43	0.030	4.4E-07	< 1E-300	Forced vital capacity (FVC), best measure
0.070	5.5E-40	0.029	5.1E-08	< 1E-300	Drive faster than motorway speed limit
0.077	3.5E-36	0.038	5.0E-10	< 1E-300	Length of working week for main job
0.068	4.0E-39	0.024	4.9E-06	< 1E-300	Weekly usage of mobile phone in last 3 months
-0.065	1.3E-30	-0.043	3.6E-14	2.2E-07	Central augmentation pressure during PWA
0.069	1.1E-41	0.015	4.1E-03	< 1E-300	Standing height
0.066	8.5E-37	0.028	7.7E-08	< 1E-300	Sitting height
0.068	2.4E-40	0.017	7.8E-04	< 1E-300	Height
-0.062	2.5E-29	-0.034	6.3E-10	6.5E-11	HDL cholesterol
-0.065	2.0E-34	0.016	1.8E-03	< 1E-300	Whole body fat mass
0.086	7.1E-35	0.018	0.013	< 1E-300	RR interval
-0.058	1.6E-29	-0.025	8.2E-07	2.8E-14	Current employment status
0.060	4.6E-30	0.024	7.6E-06	< 1E-300	Risk taking
**0.029**	**3.4E-05**	**0.079**	**1.3E-30**	**< 1E-300**	**Year ended full time education**
0.061	5.2E-32	-0.011	0.028	< 1E-300	Daytime dozing / sleeping (narcolepsy)
0.081	3.7E-31	0.018	8.8E-03	< 1E-300	PP interval
-0.058	4.3E-29	-0.019	2.0E-04	< 1E-300	Worrier / anxious feelings
-0.057	8.4E-28	-0.023	1.2E-05	2.4E-15	Nervous feelings
-0.059	7.0E-26	-0.029	2.6E-07	3.7E-12	Apolipoprotein A
0.059	7.5E-30	0.014	6.5E-03	< 1E-300	Number of vehicles in household
-0.057	2.6E-28	-0.018	3.8E-04	< 1E-300	Work/job satisfaction
0.059	1.6E-26	0.025	1.0E-05	6.7E-16	Pulse wave reflection index
0.057	1.2E-28	0.013	0.014	< 1E-300	Time spent driving
0.056	1.8E-27	-0.016	1.4E-03	< 1E-300	Distance (Euclidean) to coast
-0.078	6.2E-29	-0.013	0.072	< 1E-300	QRS num
**0.003**	**0.51**	**-0.058**	**1.5E-29**	**< 1E-300**	**Touchscreen duration**
-0.064	7.6E-29	-0.008	0.14	< 1E-300	Pulse rate, automated reading
-0.071	6.7E-24	-0.032	5.3E-06	< 1E-300	QTC interval
0.067	1.1E-21	0.039	3.7E-08	3.4E-11	Heel Broadband ultrasound attenuation, direct entry
0.055	7.6E-27	0.011	0.032	< 1E-300	Operation code (1218 - vasectomy)
**0.007**	**0.20**	**-0.058**	**3.0E-27**	**< 1E-300**	**LV stroke volume**
-0.054	1.0E-24	-0.012	0.023	< 1E-300	Platelet crit
0.055	3.2E-26	0.000	0.97	< 1E-300	Getting up in morning
-0.052	4.7E-23	-0.015	4.5E-03	< 1E-300	Platelet count
-0.056	6.4E-24	-0.011	0.052	< 1E-300	Neuroticism score
-0.050	1.3E-22	-0.007	0.16	< 1E-300	Seen doctor (GP) for nerves, anxiety, tension or depression
0.050	1.9E-22	0.001	0.79	< 1E-300	Skin colour
0.052	3.2E-22	-0.004	0.47	< 1E-300	LV end systolic volume
-0.054	2.7E-22	0.000	0.96	< 1E-300	Pulse rate

#### 3.1.3 Associations of network amplitudes with genetic phenotypes

We carried out a separate GWAS for the 21 network amplitudes using 20,381,043 SNPs as described in Material and methods. The summarised GWAS results are presented in Table 5, and separate Manhattan plots for every GWAS are provided in Fig. S6. In total, we found 18 peak associations above the standard GWAS P value threshold of $-\log_{10}P=7.5$ from the discovery sample of 22,172 participants. Applying a further Bonferroni correction to account for multiple testing across these 21 GWAS (one for each network), seven of these associations passed the corrected threshold of $-\log_{10}P=-\;\text{lo}g_{10}\left(10^{-7.5}/21\right)=8.82$.

**Table 5:** Loci significantly associated with network amplitude (bold font indicates statistical significance after Bonferroni correction across networks).

**Network index**	**Network name**	**Chr**	**Position**	**RSID**	**A1**	**A2**	**Beta**	$-\log_{10}P$	**Nearest gene**	**Location**	**GTEx eQTL**
**12**	**Motor**	**2**	**114089551**	**rs2863957**	**C**	**A**	**-0.12**	**23.97**	***PAX8***	**Intergenic**	***AC016745.3, RP11-480C16.1, CBWD2, FOXD4L1***
**13**	**Language**	**10**	**134312221**	**rs753165483**	**CACAA**	**C**	**0.076**	**13.63**	***INPP5A***	**Intergenic**	**-**
**1**	**DMN**	**10**	**96026184**	**rs11289753**	**CA**	**C**	**0.067**	**11.11**	***PLCE1***	**Intron**	**-**
**14**	**Salience**	**10**	**96009182**	**rs543302184**	**T**	**TA**	**0.062**	**9.43**	***PLCE1***	**Intron**	**-**
**20**	**DMN**	**11**	**69964074**	**rs2509142**	**T**	**C**	**-0.061**	**9.42**	***ANO1***	**Intron**	***ANO1, PPFIA1***
**8**	**Visual**	**19**	**45424351**	**rs814573**	**A**	**T**	**-0.080**	**9.34**	***APOC1***	**Intergenic**	**-**
**14**	**DMN**	**10**	**134323564**	**rs11591553**	**G**	**A**	**-0.061**	**9.16**	***INPP5A***	**Intergenic**	***LINC01165, INPP5A***
6	Fronto-parietal	10	134303568	rs773501199	GTCCC	G	0.063	8.80	*INPP5A*	Intergenic	-
10	Motor	2	114083120	rs6737318	A	G	-0.070	8.63	*PAX8*	Intergenic	*AC016745.3, RP11-480C16.1, CBWD2, FOXD4L1*
20	DMN	19	45424351	rs814573	A	T	-0.076	8.53	*APOC1*	Intergenic	-
12	Motor	4	117917153	rs35575786	C	T	-0.14	8.13	*TRAM1L1*	Intergenic	-
7	DMN	11	70002987	rs3781658	G	A	-0.057	8.11	*ANO1*	Intron	*ANO1*
16	Executive control	10	134280157	rs11596664	C	T	-0.057	8.02	*PWWP2B*	Intergenic	*INPP5A, LINC01165, RP11-432J24.5*
6	Fronto-parietal	10	96039597	rs2274224	G	C	0.056	7.97	*PLCE1*	Exon	*PLCE1-AS1, NOC3L*
3	Attention	10	96012950	rs7080472	G	T	0.056	7.93	*PLCE1*	Intron	*PLCE1-AS1, NOC3L*
21	Language	18	55536924	rs6566908	G	A	-0.056	7.93	*ATP8B1*	Intergenic	-
12	Motor	9	87336518	rs111867627	A	C	-0.121	7.87	*NTRK2*	Intron	-
15	Cerebellum	9	87242552	rs148603475	C	T	-0.095	7.6	*NTRK2*	Intergenic	-
8	Visual	7	108987486	rs848866	C	T	0.063	7.57	*FLJ00325*	Intergenic	-

The amplitude of network 12 was significantly associated with the locus rs2863957 ($-\log_{10}P=23.97$), ∼50 kb from *PAX8* and ∼100 kb from *CBWD2*, and an eQTL of *CBWD2* and *FOXD4L1* in particular. The amplitudes of networks 1 and 14 were found to be significantly associated with the variants rs11289753 ($-\log_{10}P=11.11$) and rs543302184 ($-\log_{10}P=9.43$), respectively, two indels in an intron of *PLCE1*. Network 13 and 14 amplitudes showed significant associations with rs753165483 ($-\log_{10}P=13.6$) and rs11591553 ($-\log_{10}P=9.16$), two variants located ∼30 kb and ∼40 kb from *INPP5A*, respectively. Network 20 amplitude was associated with rs2509142 ($-\log_{10}P=9.43$), in an intron – and an eQTL – of *ANO1*. Finally, rs814573, a variant less than 2kb from *APOC1*, was significantly associated with the amplitude of network 8 ($-\log_{10}P=9.34$).

### 3.2 Origins of network amplitude

So far, we have demonstrated how variations in network amplitudes across participants are closely linked to FC and to various non-imaging and genetic variables. The results suggest a potential use of network amplitudes as a valuable biomarker for health and disease in population-based research. It is important, however, to gain a better understanding of what network amplitude tells us about RSNs. Here, we provide new insights into network timeseries and amplitude, demonstrating that network amplitude mainly represents the level of temporal synchrony between the brain regions in a given network.

For each network, temporal synchrony and BOLD amplitude were defined as described in Section 2.3. Temporal synchrony was defined as the standard deviation of the new RSN timeseries generated by dual regression using voxel-wise temporally normalized fMRI data and therefore represents the degree of within-network phase synchronisation across voxels after removing raw voxel-wise signal amplitude (Fig. 1C). On the other hand, BOLD amplitude represents the *mean* BOLD fluctuation amplitudes (standard deviations) of voxels contributing strongly (|Z| > 3.29) to the network, and therefore represents “raw” within-network signal amplitude across voxels after removing synchronisation effects.

#### 3.2.1 Network amplitude is mainly due to temporal synchronisation within the RSNs

**Fig. 6:**
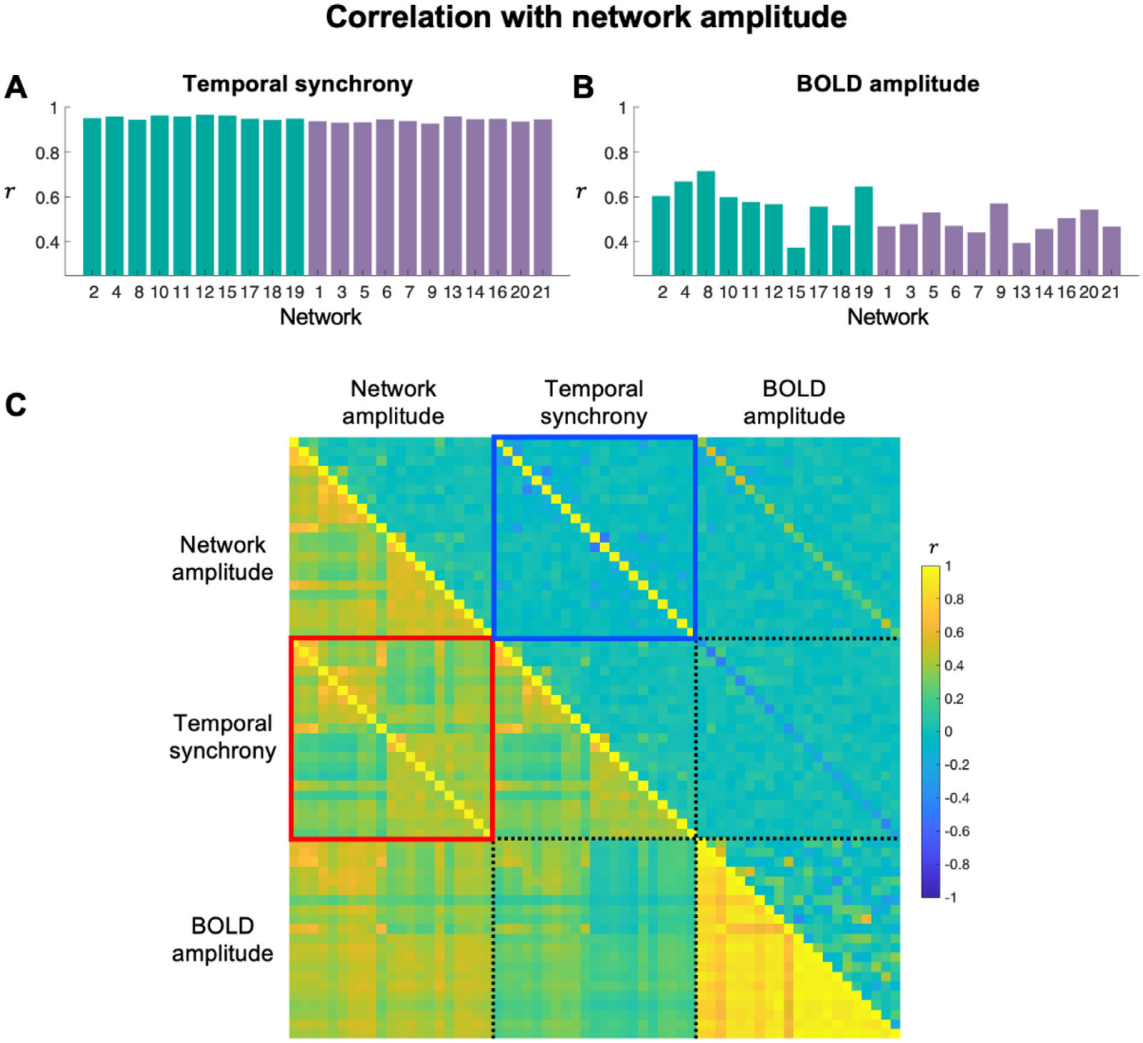
**(A)** Correlations between network amplitude and temporal synchrony across participants. **(B)** Correlations between network amplitude and BOLD amplitude across participants. The networks are color-coded such that green and purple colours represent sensory and cognitive networks, respectively, based on the clustering analysis result in Fig. 4. **(C)** Full (below diagonal) and partial (above diagonal) correlations of the network amplitudes, temporal synchrony, and BOLD amplitudes. The correlation between network amplitude and temporal synchrony is high for every network (diagonal elements in the red box) even after removing all other information (diagonal elements in the blue box). The networks are presented in the same order as in panels (A) and (B).

We found strikingly high correlations (r = 0.89 ± 0.02) between network amplitude and temporal synchrony across participants (Fig. 6A). On average, 80% of the intersubject variance of the network amplitudes was explained by temporal synchrony. In contrast, the correlations between network and BOLD amplitudes were much lower (r = 0.38 ± 0.09) (Fig. 6B), and hence approximately 16% of the intersubject variance of the network amplitudes was explained by the BOLD amplitudes. Partial correlations between network amplitude and temporal synchrony across participants (in the upper triangle of Fig. 6C) further supports the more direct link between network amplitudes and temporal synchrony, compared with the weaker link with BOLD amplitudes. The results remained very similar even if we used the BOLD amplitudes computed using the weighted-averaging methods (Fig. S3B).

These results demonstrate that temporal synchrony across voxels (within the brain regions involved in a given network) is the main determinant of the network amplitude, and the network amplitude is much less sensitive to the scale of the raw BOLD fluctuations of each voxel. This finding has significant implications for interpreting changes in network amplitude in that if, for instance, network amplitude decreases with age, it indicates that age is primarily associated with less synchronous signal fluctuations of the voxels in the network.

We further investigated whether temporal synchrony is determined more by a subset of the voxels contributing most strongly to the network or by all the voxels within the network.

The first panel in Fig. S4A shows that, when including only the voxels passing the group ICA map threshold of 3.29 ($P\left(|Z|=3.29\right)=10^{-3}$), the new RSN timeseries themselves (generated using temporally normalized fMRI data) remain highly similar (r = 0.90 ± 0.023 across the 21 networks) to the original, unthresholded timeseries. We found that the temporal correlations remain high (r = 0.87 ± 0.026 and 0.85 ± 0.031) when increasing the threshold to > 4.42 and > 5.33 (corresponding to $P\;<\;10^{-5}$ and $P\;<\;10^{-7}$, respectively), indicating that the temporal synchrony is determined by a subset of the voxels highly involved in the network.

The correlations between the participants’ original temporal synchrony and the temporal synchrony obtained from a subset of voxels passing a threshold of 3.29, 4.42, and 5.33 were r = 0.94 ± 0.021, 0.93 ± 0.029, and 0.91 ± 0.037, respectively (Fig. S2B).

Finally, the correlations between the participants’ original, unthresholded network amplitude and temporal synchrony obtained from thresholded maps remained also relatively high (r = 0.85 ± 0.036, 0.83 ± 0.043, and 0.82 ± 0.050) as the threshold increased to 3.29, 4.42 and 5.33, respectively (Fig. S4C).

#### 3.2.2 Relationship of temporal synchrony and bold amplitude with FC

As network amplitude reflects mainly temporal synchrony, and, to a lower extent, BOLD amplitude, it is likely that the correlations between the network amplitudes and FC described previously in Section 3.1.1 are driven by these two factors. We thus revisited the FC analyses and examined the within-subject correlations between the temporal synchrony and FC, and between the BOLD amplitudes and FC. In addition, correlations of FC with temporal synchrony and BOLD amplitudes across participants were examined.

**Fig. 7:**
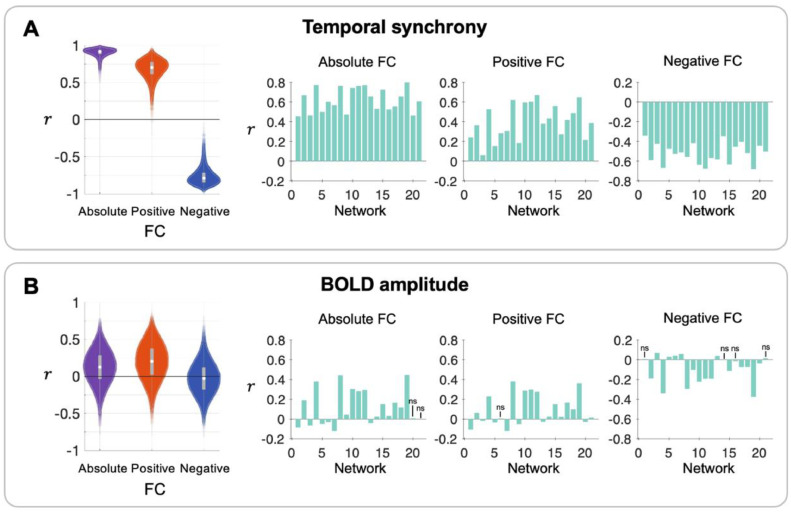
**(A)** Within-subject (across networks) correlations (left) and correlations across participants (right) between temporal synchrony and (absolute/positive/negative) FC. **(B)** Within-subject correlations (left) and correlations across participants (right) between BOLD amplitudes and (absolute/positive/negative) FC. Detailed descriptions of the plots and procedures to compute the correlation coefficients are provided in Fig. 3. Most of the P values of the correlations in (A) and (B) survived the Bonferroni correction (P_corr_ < 0.001). The few that did not survive correction are denoted as ns.

Fig. 7A shows that, similar to the results presented in Fig. 3A, there were strong within-subject correlations between the variations in the temporal synchrony and FC across the networks (absolute FC: r = 0.91 ± 0.05; positive FC: r = 0.68 ± 0.12; negative FC: r = -0.77 ± 0.10). We also found a significant (P < 0.001) correlation between temporal synchrony and FC across participants for every network and all FC types (i.e., absolute, positive, and negative).

In contrast, Fig. 7B shows that the within-subject correlations of FC with BOLD amplitudes were markedly low (absolute FC: r = 0.12 ± 0.23; positive FC: r = 0.20 ± 0.24; negative FC: r = -0.03 ± 0.21), in particular when compared with within-subject correlations obtained between the network amplitudes and FC. The correlations across participants were also much weaker overall except for the visual networks (2, 4, 8, 19) and motor networks (10, 11, 12).

The high similarity between network amplitude/FC correlations (Fig. 3A) and temporal synchrony/FC correlations (Fig. 7A) strongly suggests that the relationship between network amplitude and FC is mainly driven by the similar temporal patterns of the voxels within the networks.

#### 3.2.3 Relationship with key non-imaging variables revisited

**Fig. 8:**
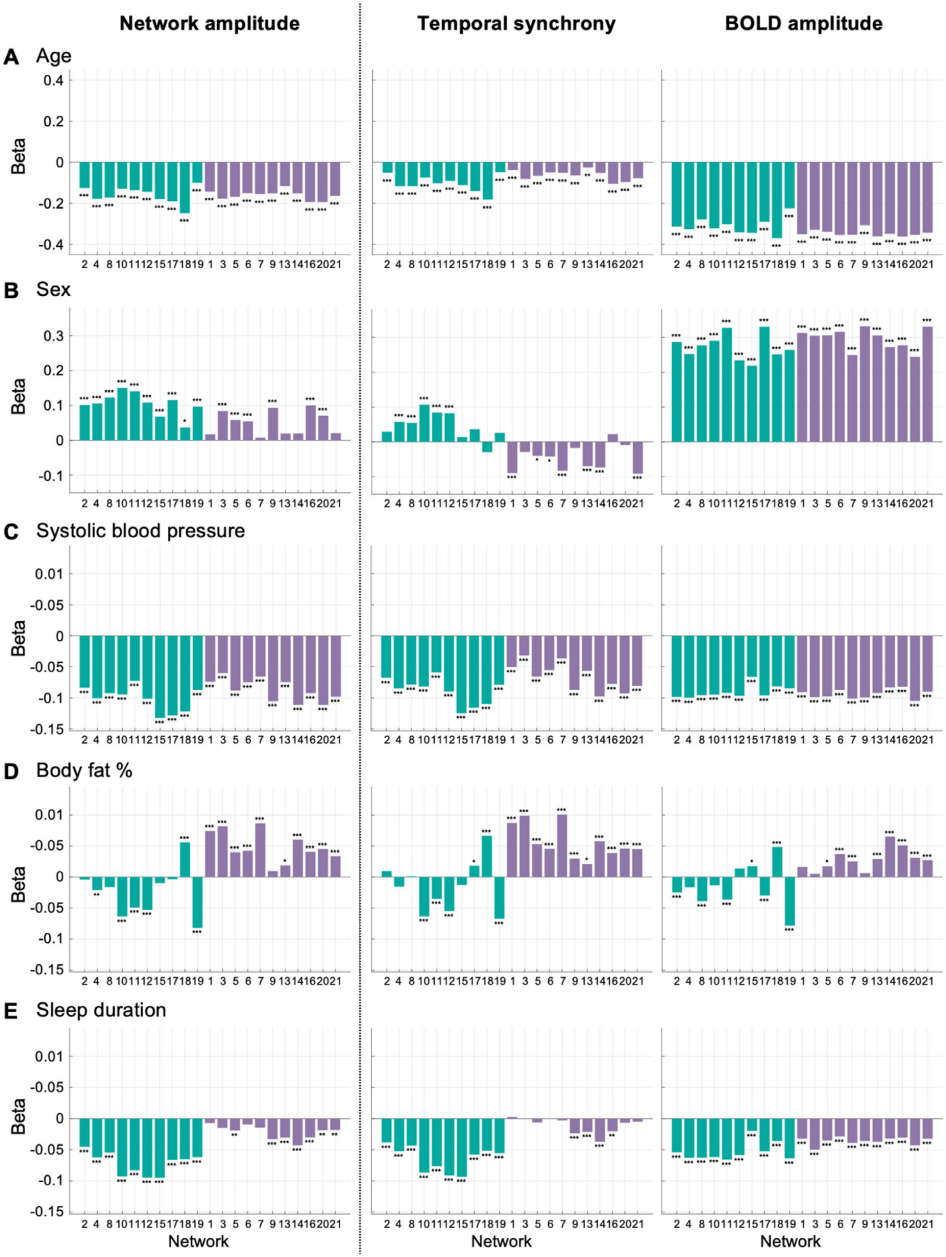
Regression coefficients (beta) estimated from the multiple linear regressions to analyze the relationship between a network amplitude/temporal synchrony/BOLD amplitude and non-imaging variables. In total, 63 multiple linear regression analyses were conducted with the same non-imaging variables (systolic blood pressure, body fat %, haemoglobin concentration, sleep duration, age, sex, age × sex, age^2^, and age^2^× sex; variables were normalized except for sex coded as 0 and 1 for female and male) as predictors. Each of the normalized network amplitudes, temporal synchrony, and BOLD amplitudes of the 21 networks was used as the dependent variable. The estimated regression coefficients from the multiple linear regression analyses are arranged according to amplitude types (columns) and predictors (rows). For brevity, only the beta coefficients for **(A)** age, **(B)** sex, **(C)** systolic blood pressure, **(D)** body fat %, and **(E)** sleep duration are presented. The networks are colour coded such that green and purple colours represent sensory and cognitive networks, respectively, based on the clustering analysis result in Fig. 4. Bonferroni-corrected P values for the beta coefficients are indicated: *: P < 0.05, **: P < 0.01, ***: P < 0.001.

As described in Section 2.11, key non-imaging variables (systolic blood pressure, body fat %, haemoglobin concentration, sleep duration, age, sex) were selected as those that are most strongly correlated with sensory and/or cognitive amplitudes. We found that both temporal synchrony and BOLD amplitudes decreased with age (Fig. 8A). The age effects on the BOLD amplitudes were relatively consistent across the networks, whereas the magnitude of age effects on the temporal synchrony was different depending on the networks. In particular, the subcortical network (18) appears to be the most sensitive to age. It can be seen from the figure that the differential age effects across the networks can be similarly found in the network amplitude results.

Male participants showed significantly higher BOLD amplitudes than female participants for all networks (Fig. 8B). On the other hand, sex effects on the temporal synchrony were the opposite for the sensory and cognitive networks: male participants had greater temporal synchrony for the sensory networks, whereas female participants had greater temporal synchrony for the cognitive networks. It is worth noting that the sex differences in the network amplitudes indicate male participants have greater amplitudes for all networks due to the higher BOLD fluctuation amplitudes, and the intriguing differential sex effects seen in temporal synchrony could be missed if looking at network amplitudes alone.

Fig. 8C shows that systolic blood pressure is negatively associated with both temporal synchrony and BOLD amplitudes of the entire networks. While the magnitude of the blood pressure effect was relatively consistent across the networks, blood pressure appeared to have differential effects on temporal synchrony.

Interestingly, body fat % had opposite effects on the sensory and cognitive networks (Fig. 8D). As the body fat % increased, temporal synchrony and BOLD amplitudes decreased for most of the sensory networks (visual and motor networks). In contrast, most of the cognitive networks showed an increase in the temporal synchrony and BOLD amplitudes as body fat % increases.

Sleep effects on the temporal synchrony also showed differences between the sensory and cognitive networks (Fig. 8E). There were greater decreases in the temporal synchrony and BOLD amplitudes for the sensory networks compared with the cognitive networks as sleep duration increases.

The regression coefficients for haemoglobin concentration, age × sex, age^2^, and age^2^ × sex are shown in Fig. S5. Haemoglobin concentration was found to be positively associated with network amplitudes, temporal synchrony, and BOLD amplitudes for all networks. In contrary, for age^2^ and interactions between age and sex (Fig. S5B–D), the effects were found to be similar between the network amplitudes and temporal synchrony, but different for the BOLD amplitudes.

#### 3.2.4 GWAS revisited

We carried out additional GWAS for the temporal synchrony and (separately) BOLD amplitude, for each network independently (i.e., 42 GWASs in total), to shed light on whether the genetic association with the amplitudes are shared with those discovered for the network amplitudes. All the results are provided as separate Manhattan plots in Fig. S6.

Table 6 lists the variants significantly associated with temporal synchrony. The results show that a large subset of the loci in Table 6 (in blue) overlap with those associated with the network amplitudes (Table 5).

**Table 6:** Loci significantly associated with temporal synchrony (bold font indicates statistical significance after Bonferroni correction). Genetic variants that overlap with those found significantly associated with network amplitude (Table 5) are in blue.

**Network index**	**Network name**	**Chr**	**Position**	**RSID**	**A1**	**A2**	**Beta**	$-\log_{10}P$	**Nearest gene**	**Location**	**GTEX eQTL**
**12**	**Motor**	**2**	**114089551**	**rs2863957**	**C**	**A**	**-0.128**	**26.81**	***PAX8***	**Intergenic**	***AC016745.3, RP11-480C16.1, CBWD2, FOXD4L1***
**13**	**Language**	**10**	**134312221**	**rs753165483**	**CACAA**	**C**	**0.071**	**12.02**	***INPP5A***	**Intergenic**	**-**
**10**	**Motor**	**2**	**114083120**	**rs6737318**	**A**	**G**	**-0.074**	**9.38**	***PAX8***	**Intergenic**	***AC016745.3, RP11-480C16.1, CBWD2, FOXD4L1***
**1**	**DMN**	**10**	**96026184**	**rs11289753**	**CA**	**C**	**0.059**	**8.85**	***PLCE1***	**Intron**	**-**
14	DMN	10	96009182	rs543302184	T	TA	0.059	8.59	*PLCE1*	Intron	-
12	Motor	4	117920021	rs35436103	C	A	-0.140	8.33	*TRAM1L1*	Intergenic	-
21	Language	18	55536924	rs6566908	G	A	-0.056	8.04	*NEDD4L*	Intergenic	-
18	Subcortical	10	96012950	rs7080472	G	T	0.057	8.02	*PLCE1*	Intron	*PLCE1-AS1, NOC3L*
17	Auditory	19	10180320	rs7359864	A	C	0.067	7.88	*C3P1*	Intergenic	*C3P1*
20	DMN	19	45424351	rs814573	A	T	-0.073	7.79	*APOC1*	Intergenic	-
14	Salience	10	134323564	rs11591553	G	A	-0.055	7.72	*INPP5A*	Intergenic	*LINC01165, INPP5A*
8	Visual	7	108987486	rs848866	C	T	0.063	7.67	*FLJ00325*	Intergenic	-
11	Motor	2	114077218	rs62158166	G	C	-0.065	7.56	*PAX8*	Intergenic	*AC016745.3, RP11-480C16.1, CBWD2, FOXD4L1*
13	Language	4	189241215	rs111786429	C	T	-0.130	7.53	*TRIML2*	Intergenic	

Table 7 shows many significant associations between BOLD amplitudes and variants near or in genes (in particular *VCAN, IFITM2, and CC2D2A*), and the variants were found to be different from those associated with network amplitudes or temporal synchrony. In fact, not a single significant locus was shared with those found for network amplitudes or temporal synchrony.

**Table 7:** Loci significantly associated with BOLD amplitudes (bold font indicates statistical significance after Bonferroni correction). Note: no genetic variant overlaps with those found significantly associated with network amplitudes (Table 5).

**Network index**	**Network name**	**Chr**	**Position**	**RSID**	**A1**	**A2**	**Beta**	$-\log_{10}P$	**Nearest gene**	**Location**	**GTEx eQTL**
**20**	**DMN**	**5**	**82741694**	**rs10058141**	**G**	**A**	**-0.072**	**12.46**	***VCAN***	**Intergenic**	***-***
**5**	**Fronto-parietal**	**5**	**82728981**	**rs9293338**	**T**	**A**	**-0.073**	**12.38**	***VCAN***	**Intergenic**	***-***
**3**	**Attention**	**5**	**82728981**	**rs9293338**	**T**	**A**	**-0.071**	**11.63**	***VCAN***	**Intergenic**	***-***
**7**	**DMN**	**5**	**82840259**	**rs309556**	**T**	**C**	**-0.125**	**11.29**	***VCAN***	**Intron**	***-***
**21**	**Language**	**5**	**82741694**	**rs10058141**	**G**	**A**	**-0.068**	**11.25**	***VCAN***	**Intergenic**	***-***
**9**	**Language**	**5**	**82742118**	**rs12188947**	**A**	**C**	**-0.067**	**10.95**	***VCAN***	**Intergenic**	***-***
**6**	**Fronto-parietal**	**5**	**82741694**	**rs10058141**	**G**	**A**	**-0.067**	**10.85**	***VCAN***	**Intergenic**	***-***
**9**	**Language**	**11**	**307808**	**rs7481219**	**A**	**G**	**0.070**	**10.67**	***IFITM2***	**Intergenic**	***-***
**13**	**Language**	**5**	**82741694**	**rs10058141**	**G**	**A**	**-0.066**	**10.46**	***VCAN***	**Intergenic**	***-***
**18**	**Subcortical**	**5**	**82741694**	**rs10058141**	**G**	**A**	**-0.066**	**10.40**	***VCAN***	**Intergenic**	***-***
**16**	**Executive Control**	**5**	**82741694**	**rs10058141**	**G**	**A**	**-0.065**	**10.20**	***VCAN***	**Intergenic**	***-***
**12**	**Motor**	**5**	**82840259**	**rs309556**	**T**	**C**	**-0.117**	**10.04**	***VCAN***	**Intron**	***-***
**2**	**Visual**	**5**	**82840259**	**rs309556**	**T**	**C**	**-0.117**	**9.92**	***VCAN***	**Intron**	***-***
**16**	**Executive Control**	**4**	**15552198**	**rs2041671**	**T**	**C**	**-0.069**	**9.77**	***CC2D2A***	**Intron**	***RP11-799M12.2, CC2D2A, FBXL5***
**6**	**Fronto-parietal**	**4**	**15552198**	**rs2041671**	**T**	**C**	**-0.069**	**9.65**	***CC2D2A***	**Intron**	***RP11-799M12.2, CC2D2A, FBXL5***
**4**	**Visual**	**5**	**82840259**	**rs309556**	**T**	**C**	**-0.113**	**9.31**	***VCAN***	**Intron**	***-***
**5**	**Fronto-parietal**	**11**	**307808**	**rs7481219**	**A**	**G**	**0.065**	**9.28**	***IFITM2***	**Intergenic**	***-***
**21**	**Language**	**4**	**15552198**	**rs2041671**	**T**	**C**	**-0.067**	**9.26**	***CC2D2A***	**Intron**	***RP11-799M12.2, CC2D2A, FBXL5***
**12**	**Motor**	**4**	**15552198**	**rs2041671**	**T**	**C**	**-0.067**	**9.21**	***CC2D2A***	**Intron**	***RP11-799M12.2, CC2D2A, FBXL5***
**10**	**Motor**	**5**	**82861251**	**rs72284621**	**TGAGA**	**T**	**-0.076**	**9.18**	***VCAN***	**Intron**	***-***
**13**	**Language**	**4**	**15552198**	**rs2041671**	**T**	**C**	**-0.067**	**9.12**	***CC2D2A***	**Intron**	***RP11-799M12.2, CC2D2A, FBXL5***
**21**	**Language**	**11**	**308290**	**rs1058900**	**T**	**C**	**0.062**	**9.11**	***IFITM2***	**Exon**	***IFITM3, RP11-326C3.13, IFITM2***
**5**	**Fronto-parietal**	**4**	**15548550**	**rs16892140**	**T**	**C**	**-0.067**	**9.08**	***CC2D2A***	**Intron**	***RP11-799M12.2, CC2D2A, FBXL5***
**15**	**Cerebellum**	**4**	**15552198**	**rs2041671**	**T**	**C**	**-0.067**	**9.06**	***CC2D2A***	**Intron**	***-***
**6**	**Fronto-parietal**	**11**	**308290**	**rs1058900**	**T**	**C**	**0.061**	**8.94**	***IFITM2***	**Exon**	***IFITM3, RP11-326C3.13, IFITM2***
**9**	**Language**	**10**	**134302745**	**rs4497325**	**G**	**A**	**0.059**	**8.90**	***INPP5A***	**Intergenic**	***INPP5A, LINC01165***
**1**	**DMN**	**5**	**82741694**	**rs10058141**	**G**	**A**	**-0.060**	**8.85**	***VCAN***	**Intergenic**	***-***
20	DMN	7	120965464	rs10668066	G	GCACC	0.069	8.80	*WNT16*	Exon	*FAM3C, WNT16, CPED1*
17	Auditory	5	82742118	rs12188947	A	C	-0.060	8.77	*VCAN*	Intergenic	*-*
11	Motor	5	82861251	rs72284621	TGAGA	T	-0.074	8.74	*VCAN*	Intron	*-*
10	Motor	4	15552198	rs2041671	T	C	-0.065	8.66	*CC2D2A*	Intron	*RP11-799M12.2, CC2D2A, FBXL5*
18	Subcortical	4	15556403	rs3822298	A	G	-0.065	8.63	*CC2D2A*	Intron	*RP11-799M12.2, CC2D2A, FBXL5*
1	DMN	4	15552198	rs2041671	T	C	-0.065	8.61	*CC2D2A*	Intron	*RP11-799M12.2, CC2D2A, FBXL5*
14	Salience	5	82741694	rs10058141	G	A	-0.059	8.55	*VCAN*	Intergenic	*-*
2	Visual	4	15552198	rs2041671	T	C	-0.064	8.53	*CC2D2A*	Intron	*RP11-799M12.2, CC2D2A, FBXL5*
3	Attention	4	15548550	rs16892140	T	C	-0.064	8.51	*CC2D2A*	Intron	*RP11-799M12.2, CC2D2A, FBXL5*
16	Executive Control	10	100134036	rs7096654	C	T	0.059	8.47	*PYROXD2*	Intergenic	*PYROXD2*
1	DMN	10	100134036	rs7096654	C	T	0.059	8.41	*PYROXD2*	Intergenic	*PYROXD2*
14	Salience	4	15552198	rs2041671	T	C	-0.063	8.14	*CC2D2A*	Intron	*RP11-799M12.2, CC2D2A, FBXL5*
7	DMN	9	32471327	rs17217231	T	C	0.110	8.14	*DDX58*	Intron	*ACO1*
16	Executive Control	11	308290	rs1058900	T	C	0.058	8.08	*IFITM2*	Exon	*IFITM3, RP11-326C3.13, IFITM2*
20	DMN	10	100176104	rs3830025	A	G	-0.065	7.96	*HPS1*	Exon	*PYROXD2*
17	Auditory	4	15593692	rs13142069	C	T	0.056	7.91	*CC2D2A*	Intron	*RP11-799M12.2*
7	DMN	12	89776284	rs770083	T	C	0.055	7.79	*DUSP6*	Intergenic	*RP11-981P6.1, POC1B-AS1, POC1B*
9	Language	13	97950019	rs9513231	T	A	0.171	7.77	*MBNL2*	Intron	*-*
3	Attention	9	32462124	rs10970989	T	C	0.106	7.75	*DDX58*	Intron	*ACO1*
3	Attention	10	100176104	rs3830025	A	G	-0.064	7.73	*HPS1*	Exon	*PYROXD2*
15	Cerebellum	20	25278464	rs111759013	A	AGTGGG	-0.055	7.67	*PYGB, ABHD12*	Exon Intron	*PYGB, ABHD12, NINL, ENTPD6, RP5-965G21.4*
13	Language	11	307808	rs7481219	A	G	0.058	7.67	*IFITM2*	Intergenic	*-*
7	DMN	4	15552198	rs2041671	T	C	-0.061	7.65	*CC2D2A*	Intron	*RP11-799M12.2, CC2D2A, FBXL5*
10	Motor	9	32454348	rs1360171	T	A	0.100	7.64	*ACO1*	Exon	*ACO1*
9	Language	10	100167436	rs45523432	T	C	-0.064	7.63	*PYROXD2*	Intron	*-*
13	Language	10	100134036	rs7096654	C	T	0.056	7.62	*PYROXD2*	Intergenic	*PYROXD2*
9	Language	9	32465289	rs10970992	T	C	0.106	7.61	*DDX58*	Intron	*ACO1*
5	Fronto-parietal	9	32465289	rs10970992	T	C	0.106	7.60	*DDX58*	Intron	*ACO1*
6	Fronto-parietal	10	100134036	rs7096654	C	T	0.056	7.57	*PYROXD2*	Intergenic	*PYROXD2*
11	Motor	4	15592864	rs7684446	C	T	0.055	7.55	*CC2D2A*	Intron	*RP11-799M12.2, CC2D2A, FBXL5*
5	Fronto-parietal	17	15074430	rs72811130	C	T	0.182	7.51	*PMP22*	Intergenic	*-*

## 4 Discussion

Temporal fluctuations of RSNs have been extensively utilized to estimate FC (functional connectivity), i.e., temporal correlations within and between functional networks. The amplitudes of these temporal fluctuations, however, have been commonly overlooked, and fundamental questions about amplitudes remain unanswered. In this work, we aimed to gain a better understanding of the amplitudes, identifying key factors that drive intersubject differences in amplitudes. In addition, we examined how variations in amplitudes are related to important demographic variability in health and disease, as well as genetic phenotypes using UKB data collected from a large cohort (N = 37,982).

We found that network amplitude largely reflects how synchronously functionally linked brain regions (within a network) activate together, and the “raw” magnitude of the regions’ activity fluctuations is relatively less important in driving the apparent network amplitudes. Approximately 80% of the intersubject variability in the network amplitudes was found to be explained by temporal synchrony, which is computed from the fMRI data after setting all voxels’ BOLD fluctuation amplitudes to one (hence removing amplitude at the voxel level). By contrast, BOLD fluctuation amplitude (which is designed to ignore changes in synchronisation across a network) was found to explain only 16% of the intersubject variability of network amplitudes. The significance of this finding is that it presents a new perspective on network amplitudes, one that is different from the common assumption of the amplitudes representing “network-level” BOLD fluctuation amplitudes. This indicates that individuals with smaller network amplitude do not necessarily have smaller BOLD fluctuation amplitudes, but rather tend to have brain regions that activate together less synchronously. Hence, network amplitudes and ALFF (which is similar to our measure of “BOLD amplitude” although ALFF may provide some denoising through the removal of incoherent high frequency signal) represent quite different properties of spontaneous fluctuations of brain activity. We emphasize that, despite the significant contribution of temporal synchrony, it should be noted that network amplitude is not entirely driven by temporal synchrony, but rather by the sum of temporal synchrony and BOLD amplitude. As prior studies demonstrated that BOLD signal variability relates to many common FC metrics (Duff et al., 2018; Garrett et al., 2013), network amplitude also reflects BOLD signal variability to some degree as demonstrated in this work (e.g., Figs. 2B and 6B).

Our finding that most variance of network amplitude is accounted for by temporal synchrony within a network brings up important points as to how network amplitude relates to so-called within-network FC. There are methodological variations in computing within-network FC, but broadly speaking, it can be assessed (1) at an ROI-ROI level by computing the pairwise correlations between the specified ROIs that comprise the network (Godwin et al., 2017; Hausman et al., 2020; Siegel et al., 2016), (2) at a voxelwise level by computing the average correlations of a voxel with the rest of all within-network voxels (Du et al., 2020), or (3) at a voxelwise level by using a dual regression spatial map obtained by multiple temporal regression of voxel timeseries against the network timeseries (Bijsterbosch et al., 2019; Nickerson et al., 2017). Network amplitude is conceptually and fundamentally different from the within-network FC obtained using the ROI-ROI approach as timeseries of every voxel in the fMRI data (as opposed to voxels within an ROI) are considered in the computation of network amplitude. For the same reason, and because a group-ICA map is utilized to weigh voxels, it is also different from the second approach. The third approach (i.e., dual regression spatial map) is presumably most relevant to network amplitude, but it would need further discussions in future studies as to how the voxelwise connectivity could be summarised into a single value and how it relates to network amplitude.

There could be several potential causes for between-subject variability in within-network temporal synchrony, including (1) the “true” variability in within-network synchrony resulting from more (or less) complex and heterogeneous functional organizations within a network, (2) changes in overlap between networks (and the interaction of this overlap – especially high in the DMN for instance (39% of its voxels) – with subject misalignment with group average network estimates), and (3) changes in noise level (e.g., more noise would make voxels appear less correlated with each other). The first case can be partially inferred from the investigation of the relationships between temporal synchrony and within-network connectivity, and this could have important clinical implications, since prior studies have shown that changes in within-network connectivity are closely associated with brain states and pathologies (Boveroux et al., 2010; Hilland et al., 2018; Li et al., 2017; von dem Hagen et al., 2013). One way to measure within-network connectivity is to use spatial maps obtained at the stage 2 of dual regression (particularly when normalising the variance of the timeseries created by stage 1, so that it is not then reflected in the stage 2 spatial maps). Evidence for variability due to differences in network overlap has been identified in prior studies (Bijsterbosch et al., 2019, 2018) showing that network-related metrics such as between-network functional connectivity are strongly influenced by the shape and exact location of functional regions of individuals. Temporal synchrony would in some sense also reflect individuals’ spatial configuration of functional brain regions, relative to the group average. Nevertheless, our result demonstrating the clear sex difference in the temporal synchrony between sensory and cognitive networks (Fig. 8B) suggests that temporal synchrony is likely not merely a product of subject misalignment.

We found that, within each participant, network amplitude is highly correlated with the mean strength of the FC that this network has with the other networks (Fig. 3). The covariations were markedly high and had a narrow distribution across participants, suggesting that this may be an intrinsic characteristic of the resting-state brain, that is consistent between different individuals. The same trend was observed with temporal synchrony (Fig. 7A), whereas the FC and BOLD fluctuation amplitudes were uncorrelated (Fig. 7B), indicating that it is the temporal synchrony of networks that has high correlation with the between-network FC strength (note: when we used full correlation FC instead of partial correlation FC, the level of FC associations in Fig. 3A and B were slightly lower overall). In line with this, it has been reported that within-subject changes in FC between the first and second scans collected from the same participants could be entirely attributed to the changes in the network amplitudes (Bijsterbosch et al., 2017). Taken together, our findings show that there is a close relationship between the temporal coherency within a network and its FC with other networks, and this warrants further research to elucidate the potential underlying causes.

Clustering networks, on the basis of the intersubject covariance structure of the network amplitudes, showed a clear separation of the networks into sensory and cognitive groups (Fig. 4). This result corresponds to the similar clustering result reported previously (Bijsterbosch et al., 2017) obtained from a different study cohort (819 HCP and 5,847 UKB participants). Fig. S7 shows that the same clustering result can be obtained from temporal synchrony, but not from BOLD fluctuation amplitudes, indicating that the hierarchical structure (where functionally related networks are clustered together) is driven by the temporal synchronisation. Our clustering result is aligned with the results from prior studies that conducted comprehensive analyses of the organization of large-scale functional networks in human. In Margulies et al. (2016), the authors used the principal gradient derived from the resting-state functional connectome matrices of 820 individuals and revealed a spectrum between unimodal and transmodal brain regions. The topography of our sensory and cognitive clusters resembles a binarized version of this unimodal-transmodal functional gradient. Similar work later confirmed that large-scale networks defined from between- and within-network functional connectivity (Schaefer et al., 2018; Yeo et al., 2011) are embedded between unimodal primary sensory and motor areas and transmodal areas serving higher-order cognition (Bazinet et al., 2021; Huntenburg et al., 2018).

Several non-imaging variables were identified to have significant between-subject associations with sensory and cognitive network amplitudes (Table 1, Table 2, Table 3, Table 4). Age was the most correlated variable, followed by cardiovascular factors, physical measures (e.g., arm fat-free mass, % body fat), and lung function factors, and their relationships with the amplitudes remained significant after controlling for age. Examining the associations with 4897 non-imaging variables for each network amplitude (Fig. S2; Table S2) instead of the sensory/cognitive cluster amplitude, age, cardiovascular factors, and physical measures tend to be most strongly associated with network amplitudes, especially for the networks clustered into the sensory group. It should be noted that, given complex interplays between demographics, physical health, and brain function, there are many possible factors underlying the relationships with the network amplitudes. It would require more advanced analyses with properly controlled potentially mediating factors to obtain more accurate interpretations of these relationships, which is outside the scope of the main objectives of the present work. Instead, we describe below some of the key non-imaging variables and discuss potential reasons for their associations with the amplitudes.

The cardiovascular variables listed in Table 1, Table 2, Table 3, Table 4 are greatly affected by vascular ageing (e.g., arterial stiffness) and important risk factors for cardiovascular diseases (Zieman et al., 2005). In this regard, the results indicate that compromised vascular health contributes in part to the decrease of the sensory and cognitive amplitudes. Numerous studies have shown poor vascular health results in deterioration of brain structure and function (Kelly and Rothwell, 2020). A meta-analysis study of blood pressure levels and brain volume has reported that 93% of the 28 studies reviewed found significant associations between high blood pressure and both global and regional brain volume reductions (Beauchet et al., 2013). Also, a recent study conducted on 616 healthy older participants (60–80 years) showed that, after controlling for covariates, higher blood pressure was still found to be associated with the decrease in volume and thickness of the grey matter covering most areas of the neocortex and cerebellum (Kharabian Masouleh et al., 2018). High blood pressure has also been implicated in increase in white matter hyperintensities (de Leeuw et al., 2002; Debette et al., 2011; Dufouil et al., 2001; Gottesman et al., 2010; Guo et al., 2009). Interestingly, the effects of high blood pressure on brain function can appear early in its course without any changes in brain structure or cognition (Naumczyk et al., 2017). It has been reported that healthy young adults who have a family history of hypertension showed significantly lower BOLD responses during a working memory task compared to those without family history of hypertension (Haley et al., 2008), indicating that some brain function can be altered in the individuals at risk before any clinical symptoms.

Forced expiratory volume (FEV) and forced vital capacity (FVC) represent the volume of air a person can exhale during a forced breath, and the total amount of air exhaled during the FEV test, respectively. They are important metrics of lung function and are known to decline with age at a faster rate in males (Thomas et al., 2019). The associations between the lung and brain have been extensively explored in the adults with chronic obstructive pulmonary disease, and it is found that reduced lung function leads to structural and functional changes in the brain, cognitive impairment, and accelerated ageing (Cook et al., 1989; Dodd, 2015; Esser et al., 2016). Notably, growing evidence points towards similar relationships between lung function and brain in healthy older adults (Emery et al., 2012). Examining 469 healthy participants aged 60–64 years, a study has found that low FEV and FVC are significant predictors for subcortical atrophy (Sachdev et al., 2006), and low FEV is additionally associated with overall brain atrophy in males. On the other hand, higher FVC was found to be positively correlated with information processing speed in females and fine motor speed in males. The implications of lung function in cognitive performance are also reported in a longitudinal study (Albert et al., 1995) that followed 1192 elderly people (70–79 years). It was found that pulmonary peak expiratory flow rate was the second-best predictor, after education level, for cognitive decline. Higher lung function is also found to relate to better cognition in children (Suglia et al., 2008), suggesting the possibility of lifespan effects of lung function on the brain.

Both sensory and cognitive network amplitudes were found to have positive relationships with lean body mass variables. In normal ageing, body composition changes due to decreased metabolic rate, resulting in reduced muscle mass and increased fat mass (St-Onge and Gallagher, 2010). We found that the relationships between lean body mass and network amplitudes remained after controlling for age, which suggests that the associations are independent of direct age effects. The relationships, therefore, may reflect effects of other common covariates such as physical activity (Bherer et al., 2013; Crespillo-Jurado et al., 2019; Spartano et al., 2019), medical conditions (e.g., hypertension) (Won et al., 2017), and lifestyles (e.g., alcohol consumption and sleep duration) (Won et al., 2017), as well as shared genetic causes (Hübel et al., 2019; Peters et al., 2020; Schnurr et al., 2016).

The types of non-imaging variables associated with either the sensory or cognitive network amplitudes appeared to often match with generally-considered sensory or cognitive traits (Table 1, Table 2, Table 3, Table 4). For instance, the cardiovascular and lung function variables are slightly, but significantly, more associated with the sensory than cognitive amplitudes. This possibly reflects that the brain's cardiovascular and respiratory regulatory mechanisms are more closely related to sensory systems (Dampney, 2016) in order to relay and process information on stimuli from the external environment (Azzalini et al., 2019). On the other hand, we found that year-ended full time education and several cognitive task scores were more strongly correlated with the cognitive amplitude. This further supports that network amplitudes contain some behaviourally meaningful information rather than being merely a by-product of some physiological and non-neural processes.

Dissociating temporal synchrony and BOLD fluctuation amplitudes reveals additional important information regarding the relationships found between the network amplitudes and non-imaging variables. The multiple regression results (Figs. 8 and S3) showed that age, blood pressure, body fat %, sleep duration, and haemoglobin concentration are associated with temporal synchrony and BOLD amplitudes similarly in terms of the direction of the effects. However, the profile of the effect sizes across various networks were markedly different, in that associations with BOLD amplitudes were relatively consistent across networks, whereas associations with temporal synchronisation were quite varied across networks.

One of the most interesting results was related to sex effects (Fig. 8B). While BOLD amplitude was consistently higher for male participants across all networks, temporal synchrony was higher for male participants only in the sensory networks and female participants showed higher temporal synchrony for cognitive networks. The majority of prior studies report sex differences in within-network FC (Allen et al., 2011; Biswal et al., 2010; Filippi et al., 2013), between-network FC (Allen et al., 2011; Filippi et al., 2013; Ritchie et al., 2018; Satterthwaite et al., 2015), and fALFF (Biswal et al., 2010) and both global and regional cerebral blood flow (CBF) (Bell et al., 2006). However, across these studies, there is a lack of consensus with respect to which functional networks exhibit sex differences and the directions of the differences. For instance, sex differences in functional network connectivity were found to be more prominent in sensory networks in (Allen et al., 2011), whereas the differences were much greater in cognitive networks in (Filippi et al., 2013). The sex differences found in this work are most similar to the results in Allen et al. (2011), where males showed greater FC within and between motor and sensory-related networks compared with females. The study also found greater low-frequency (< 0.05 Hz) BOLD signal amplitudes in males in sensorimotor and attention-related networks. However, unlike our results, they did not find sex-related differences in the other networks, possibly due to a different age group (12–71 years) and analysis methods, and a smaller sample size (N = 603). Our results are also supported by Filippi et al. (2013), which identified several brain regions with stronger FC in cognitive networks in females, compared with males. These brain regions included the cingulate cortex, dorsolateral prefrontal cortex, and inferior frontal gyrus, which have been associated with working and episodic memory (Leech and Sharp, 2014), decision-making processes (Heekeren et al., 2006), and language (Bokde et al., 2001). Future studies may wish to examine whether the differential sex effects on the temporal synchrony and BOLD amplitudes are associated with sex-related cognitive abilities (Halpern, 2012; Weiss et al., 2003).

While there was no genetic overlap between network amplitudes and BOLD amplitudes, several genetic variants were identified in both the GWAS of network amplitude and temporal synchronisation (Table 6): rs2,863,957, rs6,737,318, rs11,289,753, rs753,165,483 and rs543,302,184.

The first two of these genetic variants (rs2,863,957 and rs6,737,318) are associated with network amplitudes of motor networks #10 and #12. They are highly correlated ($r^{2}$ = 1.0) and located in intergenic regions between *CBWD2* and *PAX8*. Both loci are in particular eQTLs of *CBWD2* and *FOXD4L1* in the thyroid and associated with sleep duration in a GWAS conducted in a recent UKB study (Dashti et al., 2019). Several other variants located between *CBWD2* and *PAX8* (e.g., rs7,556,815 and rs62,158,206), also eQTLs of *CBWD2* and *FOXD4L1* in the thyroid, were found to be associated with sleep duration in UKB GWAS studies (Dashti et al., 2019; Doherty et al., 2018; Jansen et al., 2019) and the Cohorts for Heart and Aging Research in Genomic Epidemiology (CHARGE) Consortium GWAS study (Gottlieb et al., 2015). In line with these loci being associated with both sleep duration and network amplitude (and temporal synchrony), we found significant correlations between sleep duration and the network amplitudes (network 10: r = -0.10, P = 4.5E-83; network 12: r = -0.11, P = 2.2E-94) and sleep duration and temporal synchrony (network 10: r = -0.09, P = 4.9E-67; network 12: r = -0.10, P = 8.6E-82) (see also Fig. 8E). Given the close relationship between the thyroid and sleep (Green et al., 2021; Pereira and Andersen, 2014) and that the genetic variants significantly associated with both network amplitudes and temporal synchrony are modulating expression of several genes in the thyroid, our results might point at a neurophysiological origin for the association we found between network amplitude (and temporal synchrony) and sleep.

The locus rs11289753 has been reported to be significantly correlated with body fat distribution (Rask-Andersen et al., 2019) and plateletcrit (Astle et al., 2016) in recent UKB studies. It is in an intron of *PLCE1*, which is involved in lipid metabolism and regulation of immunity and inflammation (Geurts et al., 2015). Accordingly, we found significant associations between body fat % and the network amplitude (r = 0.04, P = 3.0E-16) and temporal synchrony (r = 0.09, P = 3.0E-83) of network 1 (DMN; see also Fig. 8E). This might offer a potential neurobiological mechanism underlying the associations between the DMN and obesity-related metabolic alterations (Figley et al., 2016).

The GWAS of BOLD amplitudes identified genetic variants distinctive from those described above (Table 7 and Fig. S6), most of those could be related to brain development and myelination, suggesting perhaps that the foundations of BOLD amplitudes are set very early in life. For instance, among the significant variants, two (rs309,556, rs72,284,621) are located in introns of *VCAN*, which plays a central role in brain development, synaptic plasticity and myelin repair (Elliott et al., 2018; Lau et al., 2013; Schwartz and Domowicz, 2018; Wade et al., 2013), and was found in a previous GWAS to relate to most of the white matter structural connectivity (Elliott et al., 2018). The genetic variant rs1,058,900 is located in an exon of *IFITM2*, whose role has been noted in schizophrenia (Hwang et al., 2013; Saetre et al., 2007; Volk et al., 2015). rs2,041,671 and rs16,892,140 are located in introns and eQTL of *CC2D2A*, whose expression is considerably higher in fetal brain than adult brain (Gorden et al., 2008), and whose mutation has been linked to patients with Joubert syndrome (Doherty, 2009; Gorden et al., 2008), further indicating its important role during brain development.

Taken together, the GWAS overlaps between network amplitudes and temporal synchrony provide further evidence that a “network's amplitude” largely reflects its within-network temporal synchrony. To our knowledge, our results for the first time demonstrate the differential genetic mechanisms involved in the temporal synchronisation and BOLD fluctuation amplitudes of RSNs. Further investigation should shed light on the genetic architecture of human brain function, and implications in brain disorders.

## 5 Conclusions

This work highlights that, while network amplitude reflects both temporal coherence of spontaneous fluctuations of brain regions involved in networks, and the regions’ fluctuation amplitudes, a greater emphasis should be placed on the former. Crucially, intersubject variability in network amplitude needs to be understood taking into account this temporal coherence, particularly when examining relationships with demographic, behavioural, and genetic phenotypes. For instance, we demonstrated that cognitive network amplitudes are higher in males than females due to the higher fluctuations of raw BOLD signals, and females in fact have higher temporal coherency of the cognitive networks than males (Fig. 8B). This finding would not have been discovered by looking at the network amplitudes alone. Several analytical choices such as ICA dimensions and bandpass filtering can greatly affect network amplitude estimates, and future research is warranted to investigate their effects and further validate network amplitude as a valuable metric of brain neurophysiology.

## 6 Data and code availability statement

The raw and processed imaging data, IDPs and non-imaging measures in UK Biobank are available to researchers worldwide following a data access application procedure. Data processing pipeline followed previous studies (Alfaro-Almagro et al., 2021, 2018; Miller et al., 2016), and the source codes for the pipeline can be found online (https://git.fmrib.ox.ac.uk/falmagro/UK_biobank_pipeline_v_1). The Python and MATLAB codes used in this paper are available at https://github.com/benecia2sj/networkamp.

## CRediT authorship contribution statement

**Soojin Lee:** Conceptualization, Methodology, Validation, Formal analysis, Investigation, Visualization, Writing – original draft, Writing – review & editing, Visualization. **Janine D. Bijsterbosch:** Conceptualization, Methodology, Investigation, Validation, Writing – review & editing. **Fidel Alfaro Almagro:** Methodology, Software, Data curation, Writing – review & editing. **Lloyd Elliott:** Methodology, Software, Data curation, Writing – review & editing. **Paul McCarthy:** Methodology, Software, Data curation, Writing – review & editing. **Bernd Taschler:** Investigation, Writing – review & editing. **Roser Sala-Llonch:** Conceptualization, Validation, Writing – review & editing. **Christian F. Beckmann:** Conceptualization, Methodology, Validation, Writing – review & editing. **Eugene P. Duff:** Conceptualization, Methodology, Validation, Writing – review & editing. **Stephen M. Smith:** Conceptualization, Methodology, Validation, Formal analysis, Investigation, Data curation, Writing – original draft, Writing – review & editing, Visualization, Supervision, Project administration, Funding acquisition. **Gwenaëlle Douaud:** Conceptualization, Methodology, Validation, Formal analysis, Investigation, Data curation, Writing – original draft, Writing – review & editing, Visualization, Supervision, Project administration, Funding acquisition.

## Declaration of Competing Interest

The authors declare that there is no conflict of interests regarding the publication of this paper.

## Data Availability

Data will be made available on request.

## Appendix Supplementary materials

Image, application 1

Image, application 2
