# Supplementary material for: Amplitudes of resting-state functional networks – investigation into their correlates and biophysical properties
Source: Neuroimage. 2023 Jan;265:119779. doi: 10.1016/j.neuroimage.2022.119779 (PMC10933815; doi:10.1016/j.neuroimage.2022.119779)
Supplement: Supplementary file 1 [file mmc1.docx]

# Supplementary Materials

**Table S1.** List of the selected confounding variables

| **Confound family** | **Confound group** | **UKB ID** | **# of**  **Confounds** | **Confound name** |
| --- | --- | --- | --- | --- |
| Subject | Head size | 25000 | 9 | HeadSize_Site_1  HeadSize_Site_2  HeadSize_Site_3  HeadSize_Site_1_squared  HeadSize_Site_2_squared  HeadSize_Site_3_squared  HeadSize_Site_1_inormal  HeadSize_Site_3_inormal  HeadSize_Site_3_squared_inormal |
| Motion | Head motion | - | 36 | HeadMotion_mean_rfMRI_abs_Site_1  HeadMotion_median_rfMRI_abs_Site_1  HeadMotion_p90_rfMRI_abs_Site_1  HeadMotion_mean_rfMRI_rel_Site_1  HeadMotion_median_rfMRI_rel_Site_1  HeadMotion_p90_rfMRI_rel_Site_1  HeadMotion_mean_tfMRI_abs_Site_1  HeadMotion_median_tfMRI_abs_Site_1  HeadMotion_p90_tfMRI_abs_Site_1  HeadMotion_mean_tfMRI_rel_Site_1  HeadMotion_median_tfMRI_rel_Site_1  HeadMotion_p90_tfMRI_rel_Site_1  HeadMotion_mean_rfMRI_abs_Site_2  HeadMotion_median_rfMRI_abs_Site_2  HeadMotion_p90_rfMRI_abs_Site_2  HeadMotion_mean_rfMRI_rel_Site_2  HeadMotion_median_rfMRI_rel_Site_2  HeadMotion_p90_rfMRI_rel_Site_2  HeadMotion_mean_tfMRI_abs_Site_2  HeadMotion_median_tfMRI_abs_Site_2  HeadMotion_p90_tfMRI_abs_Site_2  HeadMotion_mean_tfMRI_rel_Site_2  HeadMotion_median_tfMRI_rel_Site_2  HeadMotion_p90_tfMRI_rel_Site_2  HeadMotion_mean_rfMRI_abs_Site_3  HeadMotion_median_rfMRI_abs_Site_3  HeadMotion_p90_rfMRI_abs_Site_3  HeadMotion_mean_rfMRI_rel_Site_3  HeadMotion_median_rfMRI_rel_Site_3  HeadMotion_p90_rfMRI_rel_Site_3  HeadMotion_mean_tfMRI_abs_Site_3  HeadMotion_median_tfMRI_abs_Site_3  HeadMotion_p90_tfMRI_abs_Site_3  HeadMotion_mean_tfMRI_rel_Site_3  HeadMotion_median_tfMRI_rel_Site_3  HeadMotion_p90_tfMRI_rel_Site_3 |
| Table | Scan position | 25756  25757  25758  25759 | 21 | TablePos_COG_X_Site_1  TablePos_COG_Y_Site_1  TablePos_COG_Z_Site_1  TablePos_Table_Site_1  TablePos_COG_X_Site_2  TablePos_COG_Y_Site_2  TablePos_COG_Z_Site_2  TablePos_Table_Site_2  TablePos_COG_X_Site_3  TablePos_COG_Y_Site_3  TablePos_COG_Z_Site_3  TablePos_Table_Site_3  TablePos_COG_Y_Site_1_inormal  TablePos_COG_Z_Site_1_squared  TablePos_COG_Z_Site_2_squared  TablePos_COG_Z_Site_3_squared  TablePos_Table_Site_1_squared  TablePos_COG_Z_Site_1_inormal  TablePos_COG_Z_Site_3_inormal  TablePos_COG_Z_Site_1_squared_inormal  TablePos_COG_Z_Site_3_squared_inormal |
| Acquisition | Site | 54 | 59 | ACQT_Site_1__01  ACQT_Site_1__02  ACQT_Site_1__03  ACQT_Site_1__04  ACQT_Site_1__05  ACQT_Site_1__06  ACQT_Site_1__07  ACQT_Site_1__08  ACQT_Site_1__09  ACQT_Site_1__10  ACQT_Site_1__11  ACQT_Site_1__12  ACQT_Site_1__13  ACQT_Site_1__14  ACQT_Site_1__15  ACQT_Site_1__16  ACQT_Site_1__17  ACQT_Site_1__18  ACQT_Site_2__01  ACQT_Site_2__02  ACQT_Site_2__03  ACQT_Site_2__04  ACQT_Site_2__05  ACQT_Site_2__06  ACQT_Site_2__07  ACQT_Site_2__08  ACQT_Site_2__09  ACQT_Site_2__10  ACQT_Site_2__11  ACQT_Site_2__12  ACQT_Site_2__13  ACQT_Site_2__14  ACQT_Site_2__15  ACQT_Site_2__16  ACQT_Site_2__17  ACQT_Site_2__18  ACQT_Site_2__19  ACQT_Site_2__20  ACQT_Site_3__01  ACQT_Site_3__02  ACQT_Site_3__03  ACQT_Site_3__04  ACQT_Site_3__05  ACQT_Site_3__06  ACQT_Site_3__07  ACQT_Site_3__08  ACQT_Site_3__09  ACQT_Site_3__10  ACQT_Site_3__11  ACQT_Site_3__12  ACQT_Site_3__13  ACQT_Site_3__14  ACQT_Site_3__15  ACQT_Site_3__16  ACQT_Site_3__17  ACQT_Site_3__18  ACQT_Site_3__19  ACQT_Site_3__20  ACQT_Site_3__21 |
| Time | Acquisition time | 53 | 59 | DATE_Site_1__01  DATE_Site_1__02  DATE_Site_1__03  DATE_Site_1__04  DATE_Site_1__05  DATE_Site_1__06  DATE_Site_1__07  DATE_Site_1__08  DATE_Site_1__09  DATE_Site_1__10  DATE_Site_1__11  DATE_Site_1__12  DATE_Site_1__13  DATE_Site_1__14  DATE_Site_1__15  DATE_Site_1__16  DATE_Site_1__17  DATE_Site_1__18  DATE_Site_2__01  DATE_Site_2__02  DATE_Site_2__03  DATE_Site_2__04  DATE_Site_2__05  DATE_Site_2__06  DATE_Site_2__07  DATE_Site_2__08  DATE_Site_2__09  DATE_Site_2__10  DATE_Site_2__11  DATE_Site_2__12  DATE_Site_2__13  DATE_Site_2__14  DATE_Site_2__15  DATE_Site_2__16  DATE_Site_2__17  DATE_Site_2__18  DATE_Site_2__19  DATE_Site_2__20  DATE_Site_3__01  DATE_Site_3__02  DATE_Site_3__03  DATE_Site_3__04  DATE_Site_3__05  DATE_Site_3__06  DATE_Site_3__07  DATE_Site_3__08  DATE_Site_3__09  DATE_Site_3__10  DATE_Site_3__11  DATE_Site_3__12  DATE_Site_3__13  DATE_Site_3__14  DATE_Site_3__15  DATE_Site_3__16  DATE_Site_3__17  DATE_Site_3__18  DATE_Site_3__19  DATE_Site_3__20  DATE_Site_3__21 |
| **Total** |  |  | **184** |  |

**Table S2**. List of top 100 non-imaging variables that are most significantly associated with each of the 21 network amplitudes. Unadjusted P values are displayed.

Please see the spreadsheets in **supplementary_table2.xlsx**.


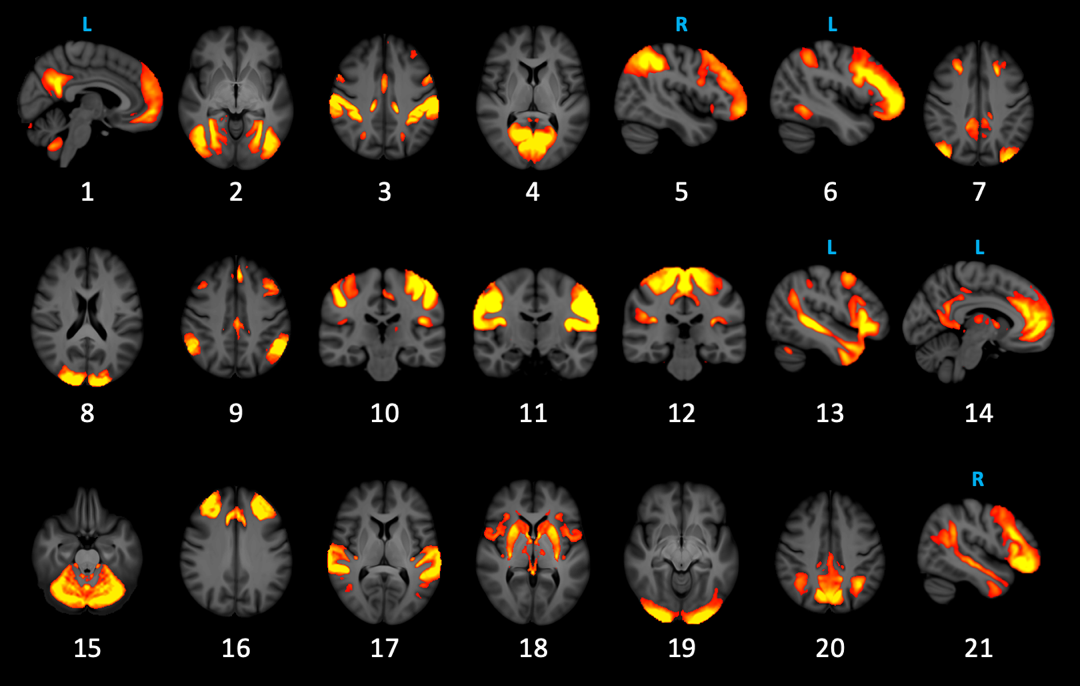


**Fig. S1**. Maps of the 21 group ICA components (networks), showing one representative slice for each. Left (L) or right (R) hemisphere is indicated in blue colour in the sagittal slices. Axial and coronal slices are displayed in radiological convention (i.e., right of the subject on the left of the figure).


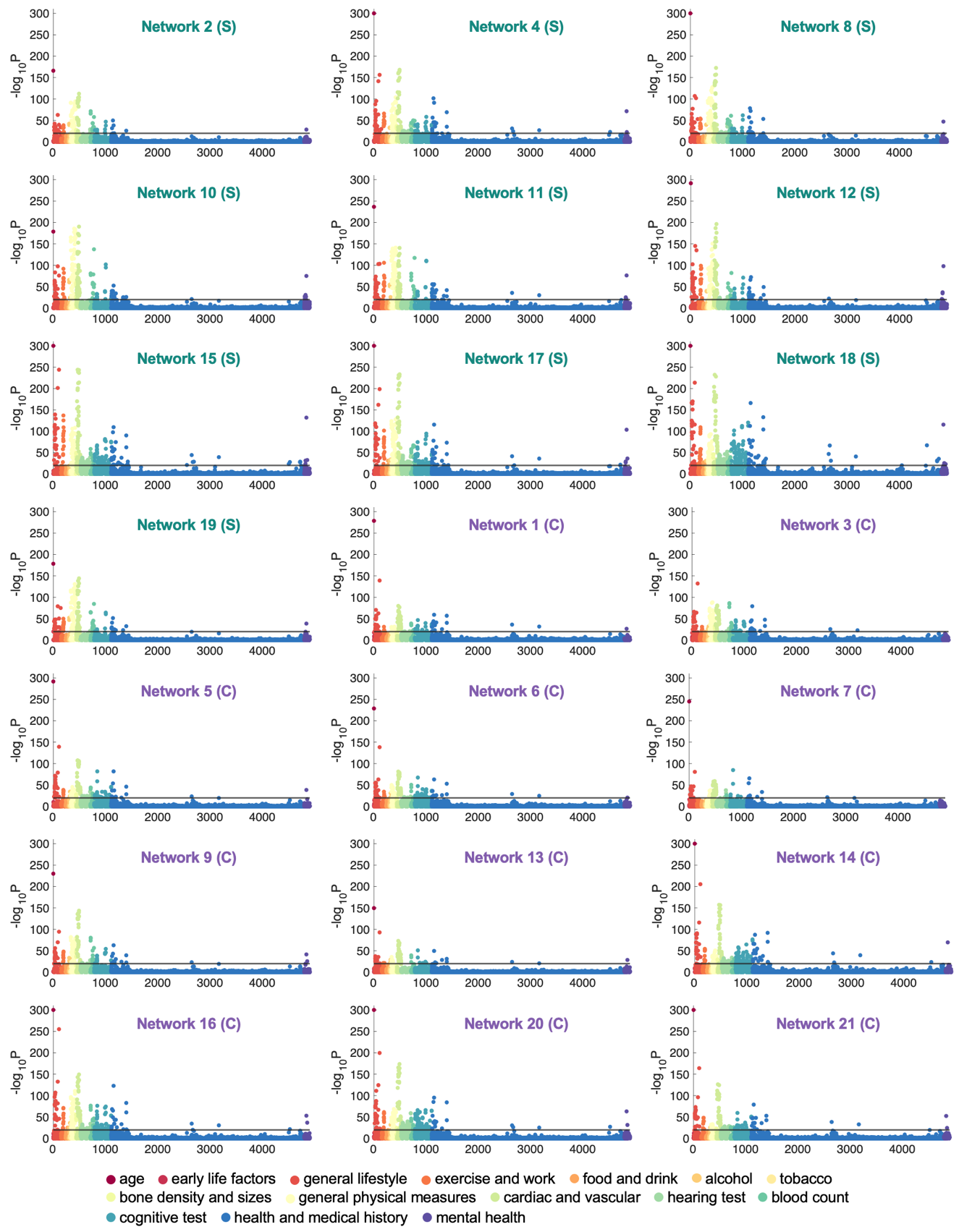


**Fig. S2.** Manhattan plots showing the associations between 4.897 non-imaging variables and 21 network amplitudes. The association strengths are presented as the Pearson correlation $P$ values that have been converted to $-{log}_{10} P$. The horizontal lines indicate $-{log}_{10} P =20$. Networks are ordered and colour coded according to the sensory (green) and cognitive (purple) clusters shown in Fig. 4.


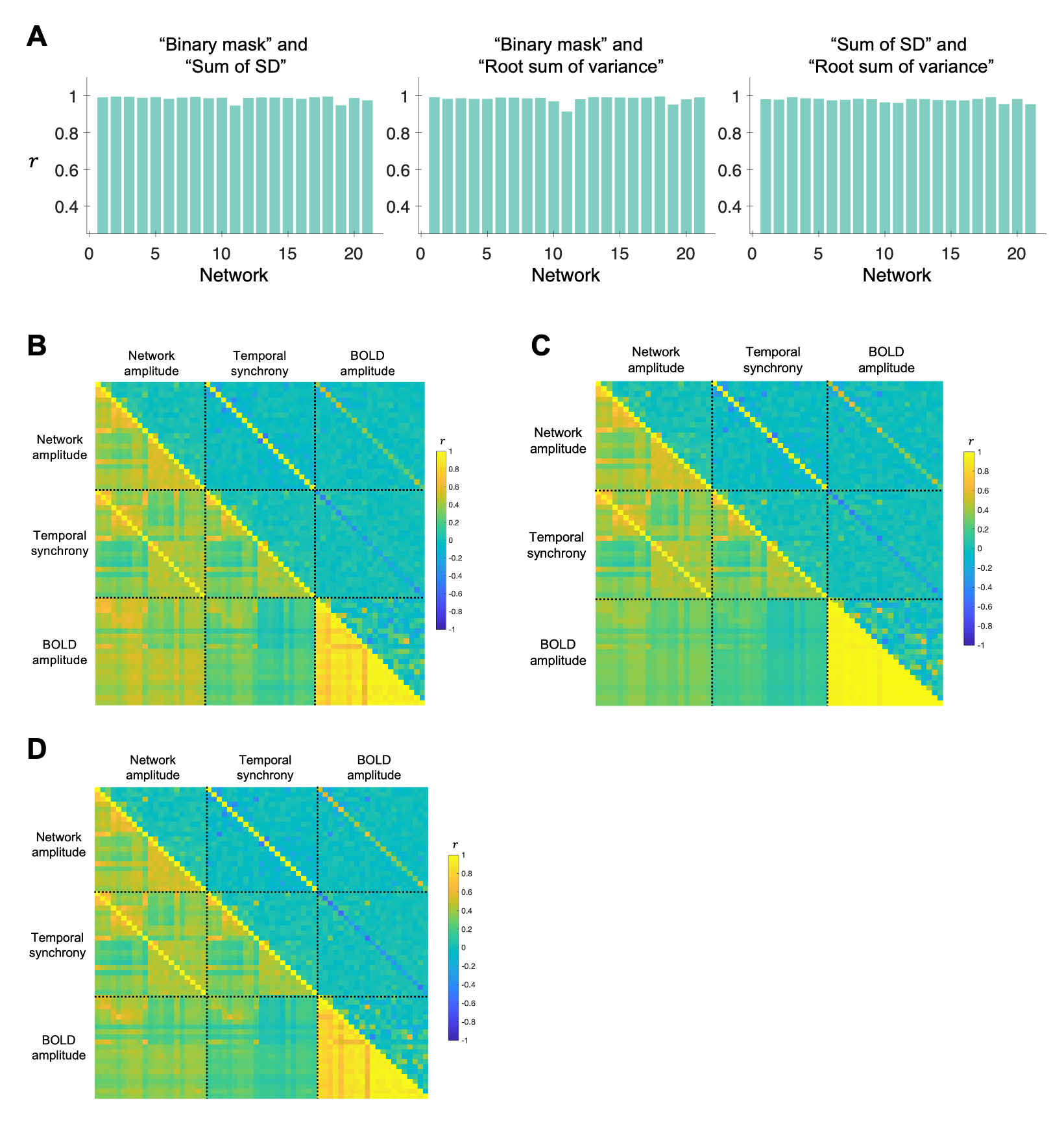


**Fig. S3.** Comparison of BOLD amplitudes computed in three different methods (binary mask: $\dot{a}_{k}={\frac{1}{\sum_{i=1}^{N} m_{k}(i)}m}_{k}^{T}v$; sum of standard deviation (SD): $\dot{a}_{k}={\frac{1}{N}g}_{k}^{T}v$; root sum of variance: $\dot{a}_{k}=\sqrt{\left( g_{k}\circ g_{k} \right)^{T}(v\circ v)}$). Mathematical notations are described in detail in Section 2.3.3. **(A)** Pairwise correlations between BOLD amplitudes computed using the three different methods. **(B–D)** Full (below diagonal) and partial (above diagonal) correlations of the network amplitudes, temporal synchrony, and BOLD amplitudes. The BOLD amplitudes were computed using the binary mask **(B)**, sum of SD **(C)**, or root sum of variance **(D)** methods. Note that **(B)** is the same results presented in Fig. 6C.


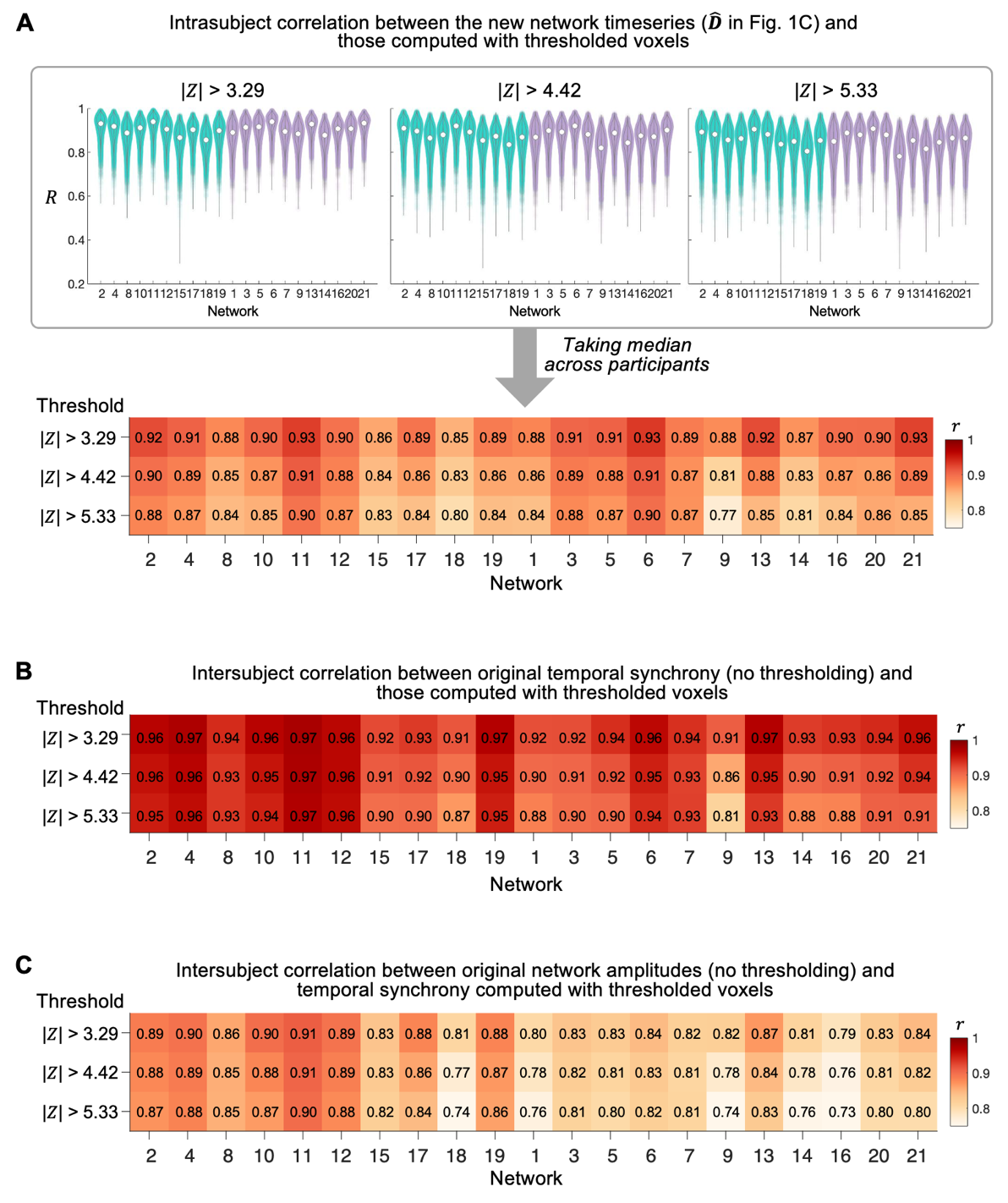


**Fig. S4**. **(A)** Distribution of temporal correlations between the new network timeseries (i.e., $\hat{\boldsymbol{D}}$ in Fig. 1C) computed using all the voxels (no thresholding) and those computed using the voxels passing the threshold shown at the top. The correlation coefficients were computed within each participant. The violin plots show the distribution of the correlation coefficients computed from 37,842 participants. The median values of the distributions are visualized below in a heatmap. **(B)** The heatmap demonstrates intersubject correlations between the original temporal synchrony (no thresholding) and those computed using the thresholded voxels. The correlations were computed for each network with varying thresholds denoted on the left. **(C)** The heatmap shows intersubject correlations between the network amplitudes (no thresholding) and temporal synchrony computed using the thresholded voxels.


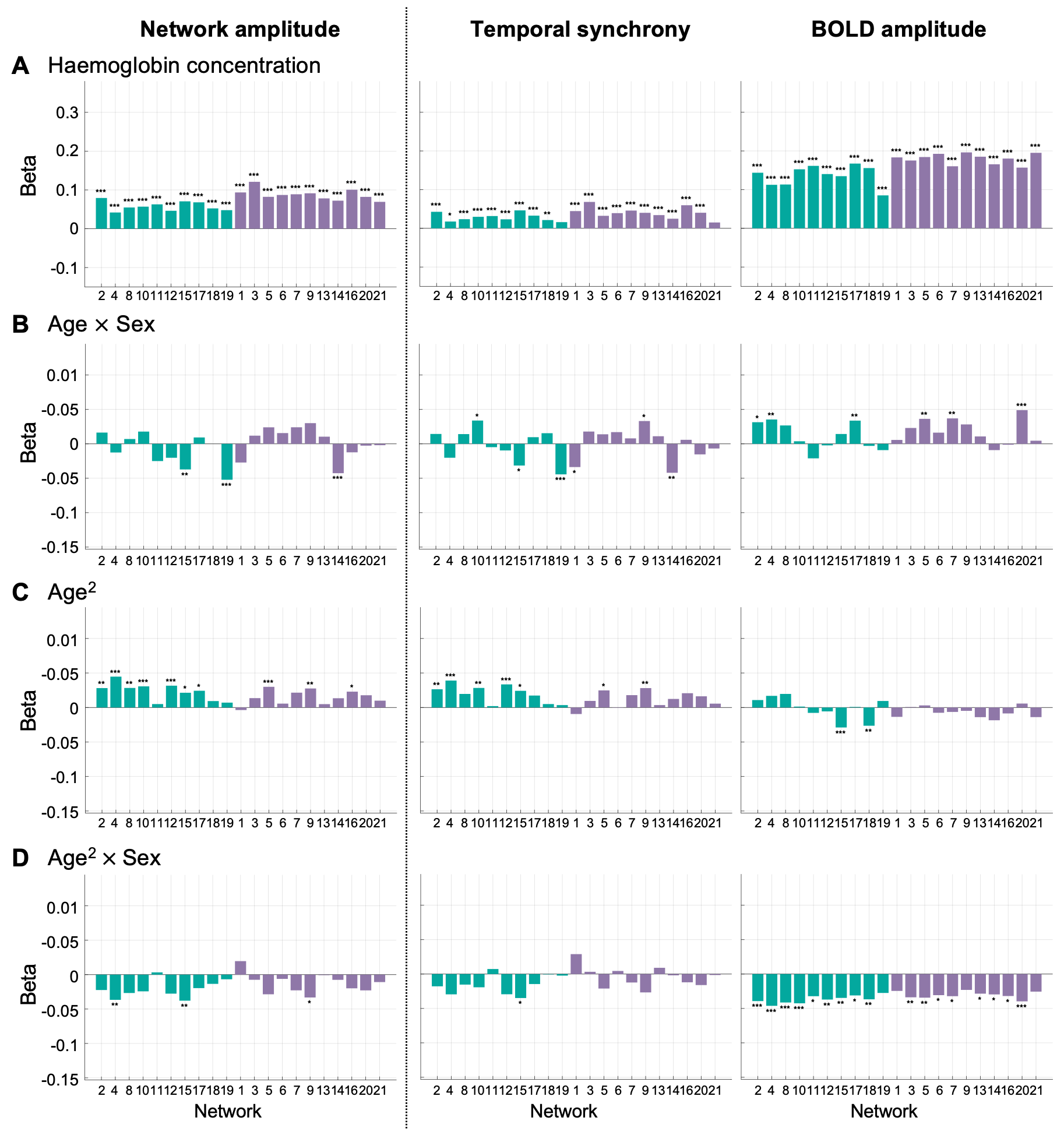


**Fig. S5**. Regression coefficients estimated from the multiple linear regressions. **(A)** Haemoglobin concentration, **(B)** age $\times$ sex, **(C)** age^2^, **(D)** age^2^ $\times$ sex. Detailed descriptions on the linear regression model and visualization are provided in Fig. 8.


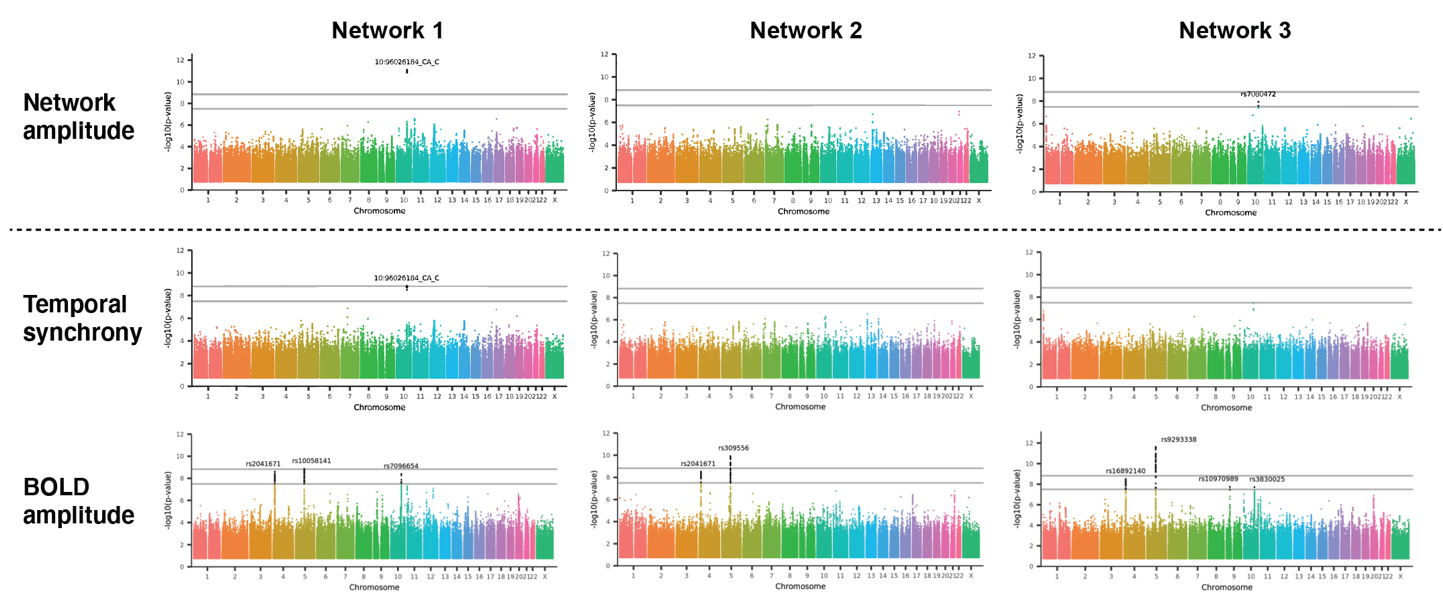

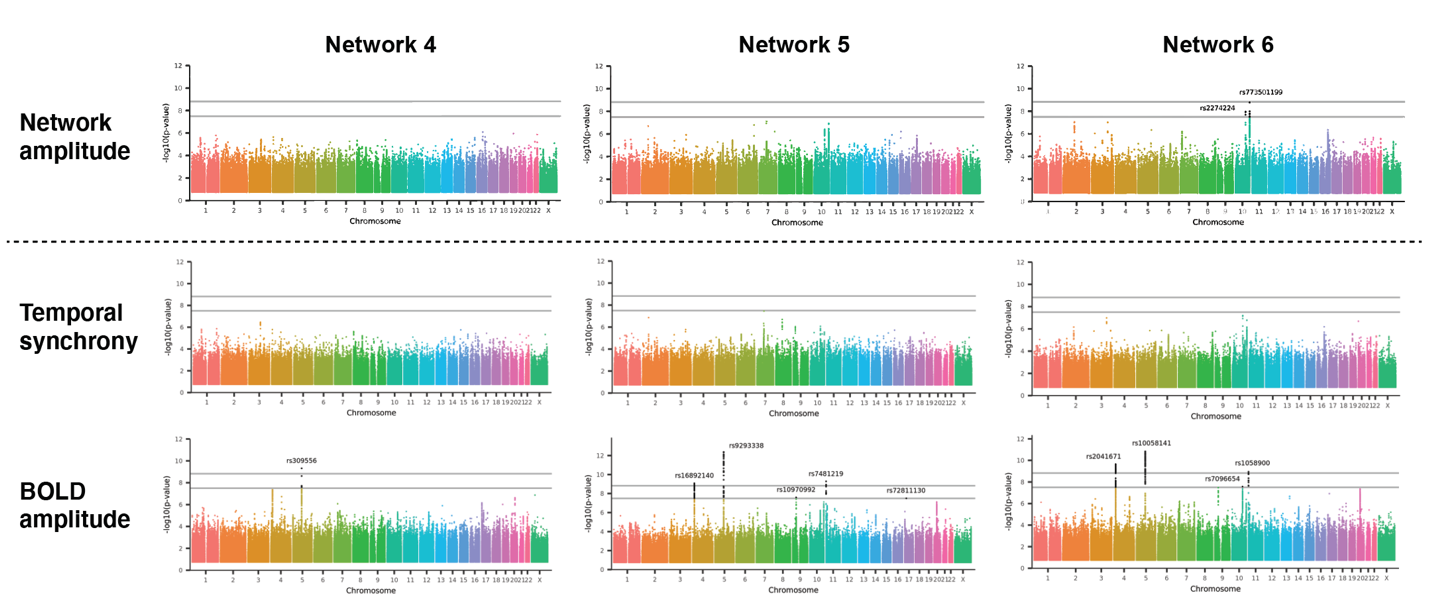


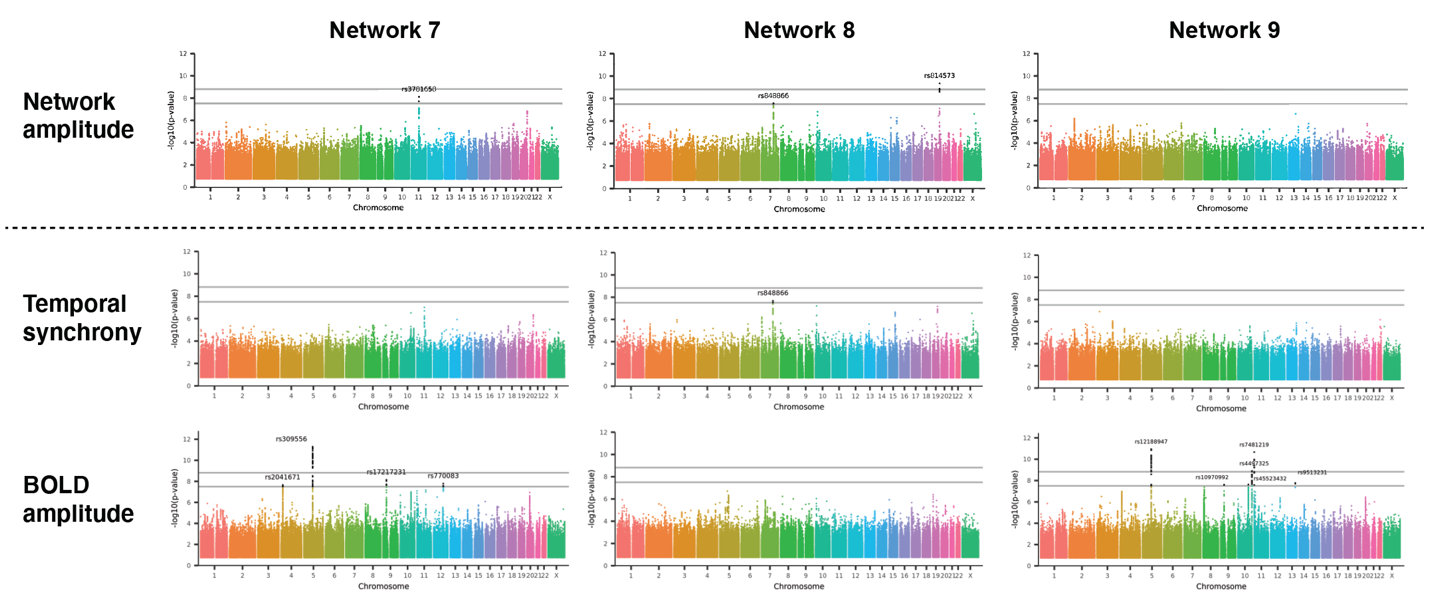


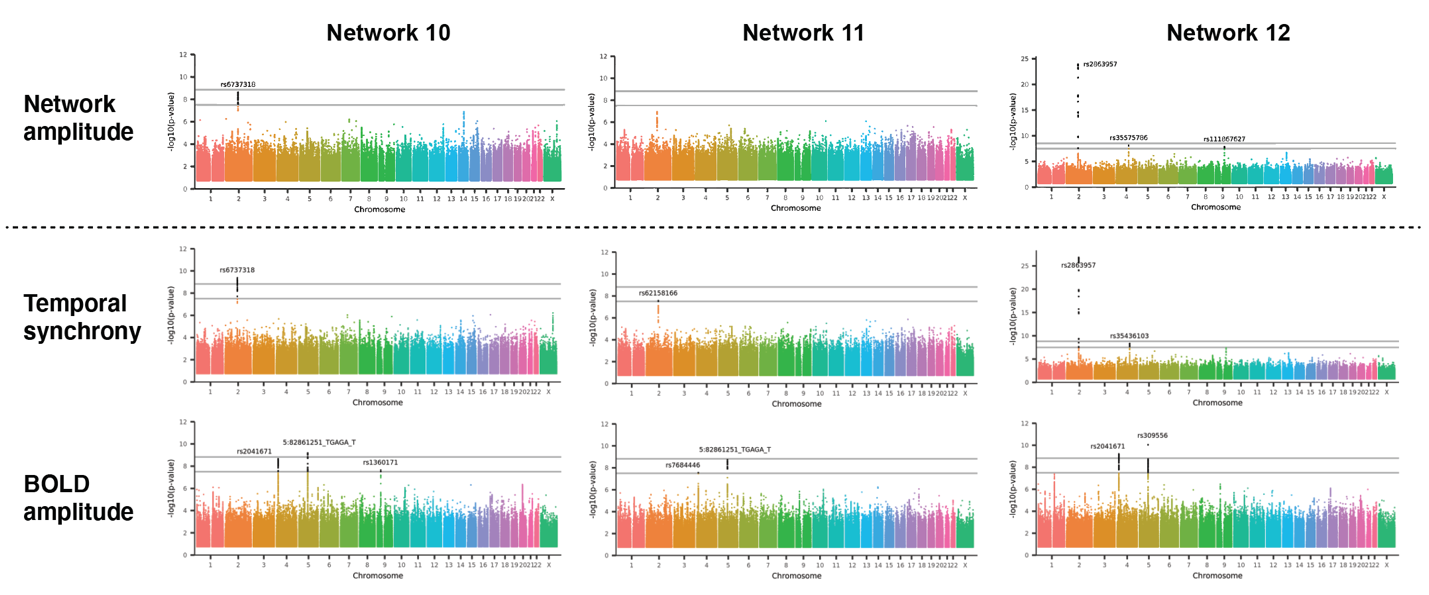


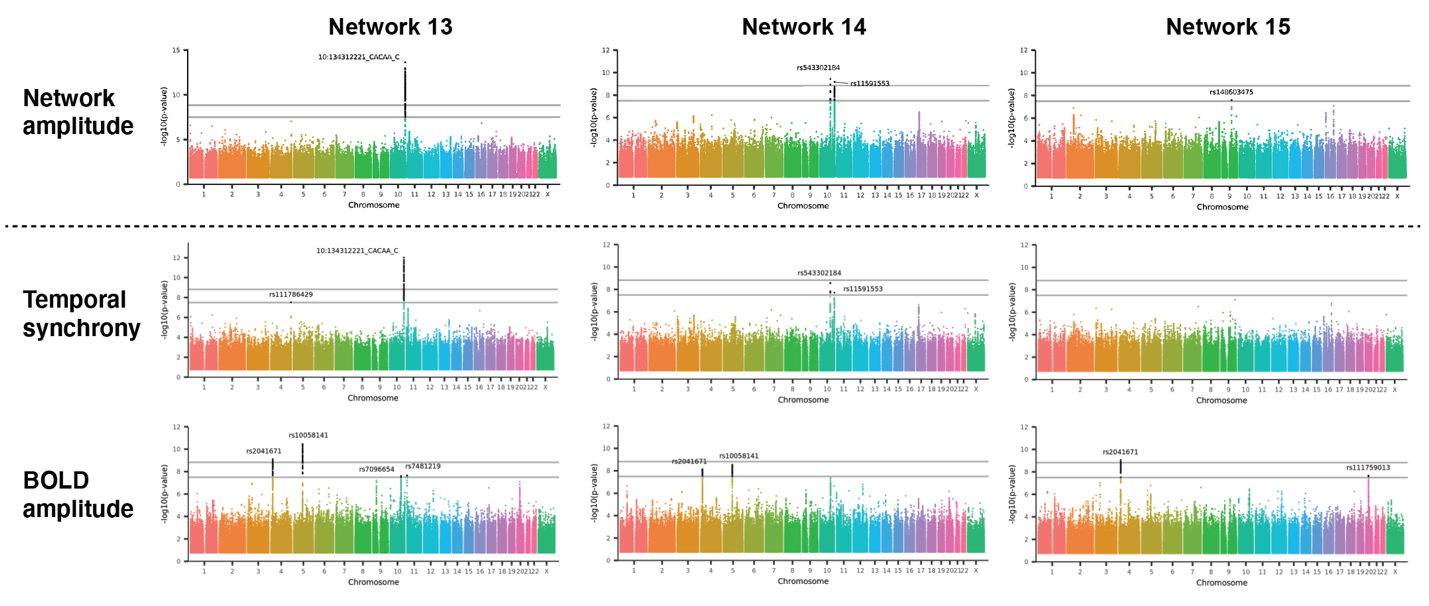


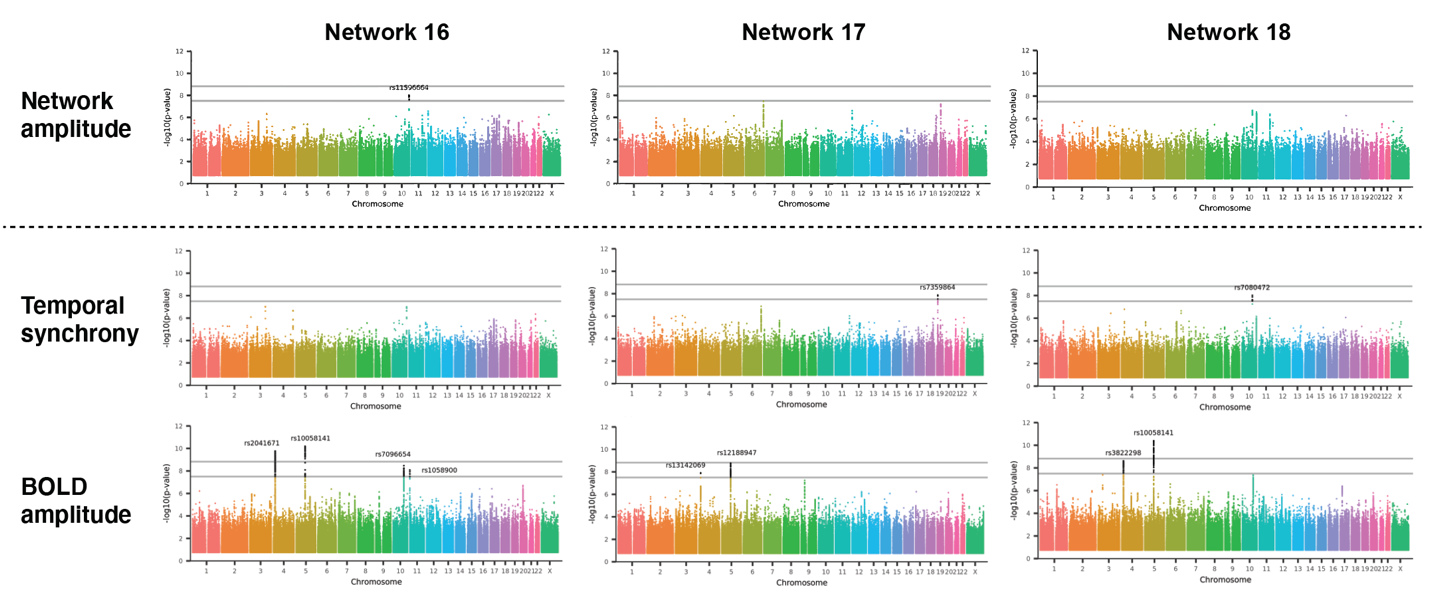


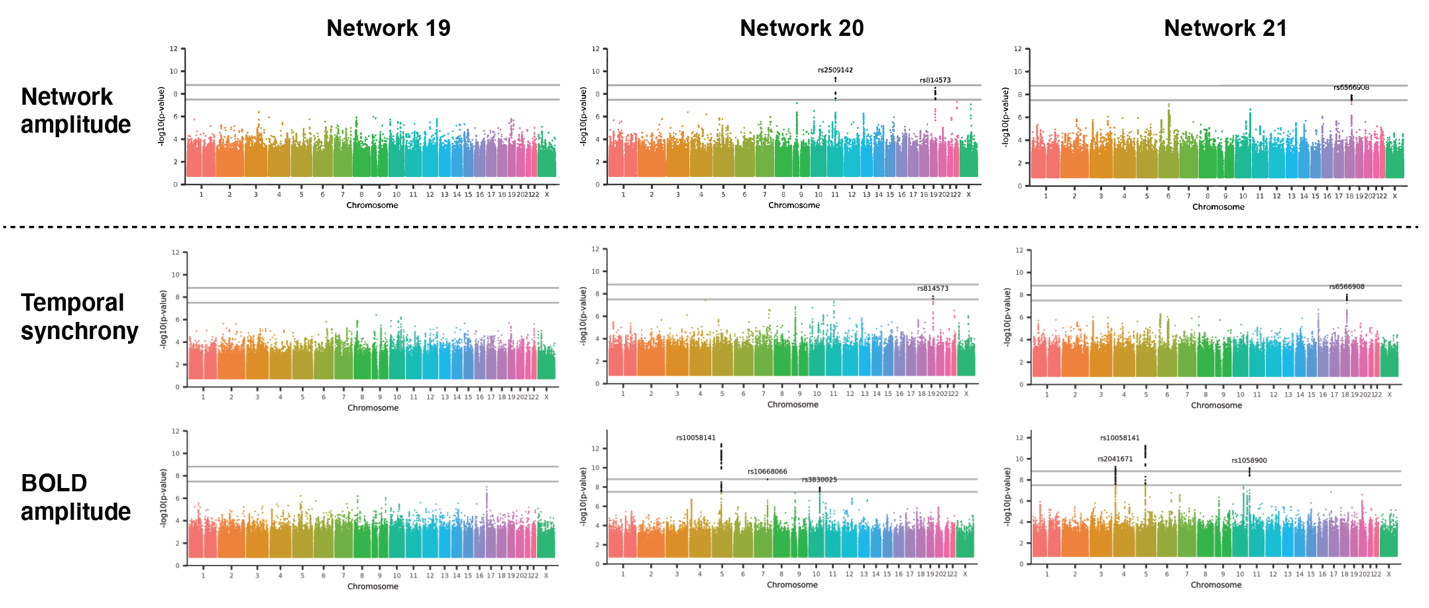


**Fig. S6.** Manhattan plots of GWASs of network amplitudes, temporal synchrony, and BOLD amplitudes. The genetic variants significantly associated with network amplitude and temporal synchrony are similar to each other while they are distinct from those associated with BOLD amplitudes.


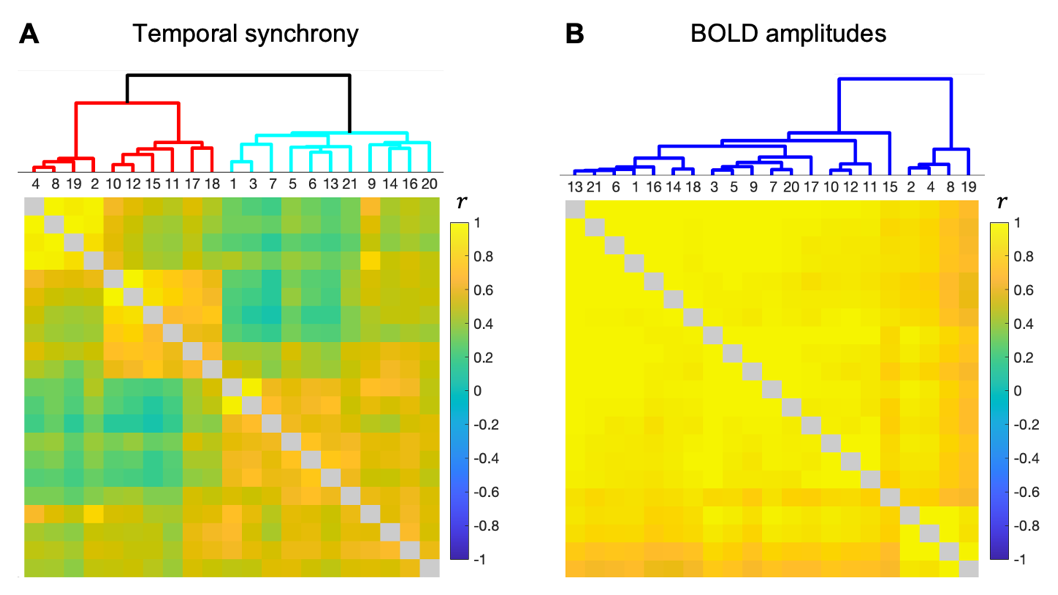


**Fig. S7**. Ward’s clustering results obtained from the cross-subject correlation matrix ($\in\mathbb{R}^{21\times21}$) computed from **(A)** temporal synchrony matrix ($\in\mathbb{R}^{\mathrm{participant}\times21}$) and **(B)** BOLD amplitude matrix ($\in\mathbb{R}^{\mathrm{participant}\times21}$). A clear separation of the sensory networks (red) and cognitive networks (cyan) is shown in (A), whereas most of the nodes are highly similar with each other in (B).
